# A conserved oligomerization domain in the disordered linker of coronavirus nucleocapsid proteins

**DOI:** 10.1126/sciadv.adg6473

**Published:** 2023-04-05

**Authors:** Huaying Zhao, Di Wu, Sergio A. Hassan, Ai Nguyen, Jiji Chen, Grzegorz Piszczek, Peter Schuck

**Affiliations:** ^1^ Laboratory of Dynamics of Macromolecular Assembly, National Institute of Biomedical Imaging and Bioengineering, National Institutes of Health, Bethesda, MD 20892, USA.; ^2^ Biophysics Core Facility, National Heart, Lung, and Blood Institute, National Institutes of Health, Bethesda, MD 20892, USA.; ^3^ Bioinformatics and Computational Biosciences Branch, National Institute of Allergy and Infectious Diseases, National Institutes of Health, Bethesda, MD 20892, USA.; ^4^ Advanced Imaging and Microscopy Resource, National Institute of Biomedical Imaging and Bioengineering, National Institutes of Health, Bethesda, MD 20892, USA.

## Abstract

The nucleocapsid (N-)protein of severe acute respiratory syndrome coronavirus 2 (SARS-CoV-2) has a key role in viral assembly and scaffolding of the viral RNA. It promotes liquid-liquid phase separation (LLPS), forming dense droplets that support the assembly of ribonucleoprotein particles with as-of-yet unknown macromolecular architecture. Combining biophysical experiments, molecular dynamics simulations, and analysis of the mutational landscape, we describe a heretofore unknown oligomerization site that contributes to LLPS, is required for the assembly of higher-order protein-nucleic acid complexes, and is coupled to large-scale conformational changes of N-protein upon nucleic acid binding. The self-association interface is located in a leucine-rich sequence of the intrinsically disordered linker between N-protein folded domains and formed by transient helices assembling into trimeric coiled-coils. Critical residues stabilizing hydrophobic and electrostatic interactions between adjacent helices are highly protected against mutations in viable SARS-CoV-2 genomes, and the oligomerization motif is conserved across related coronaviruses, thus presenting a target for antiviral therapeutics.

## Teaser

The oligomerization site is key for ribonucleoprotein assembly and presents a new antiviral target for SARS-CoV-2.

## INTRODUCTION

Intense research effort is devoted to understand the pathology and mechanisms of the severe acute respiratory syndrome coronavirus 2 (SARS-CoV-2) virus, which infects on the order of 1 million people daily. While highly effective vaccines and therapeutics have been developed in short time, continued viral evolution is a major concern that motivates the development of new broadly effective and multipronged approaches (*1*). For example, substantial work is devoted to understand the structural implications of spike protein mutations and their impact on receptor binding and immune evasion and, similarly, of emerging escape mutants for viral protease inhibitors (*2*, *3*). The focus of the present work is the nucleocapsid (N)-protein, which is the most abundant viral protein in virus infected cells (*4*) with an estimated 1% of total protein (*5*). Despite its role as a key structural protein for viral assembly, presenting as a major antigen, and being the locus of mutations that can enhance viral assembly and/or infectivity (*6*–*10*), it is still poorly understood in its many functions, and as of yet not a target of successfully deployed therapeutics.

**Fig. 1.**
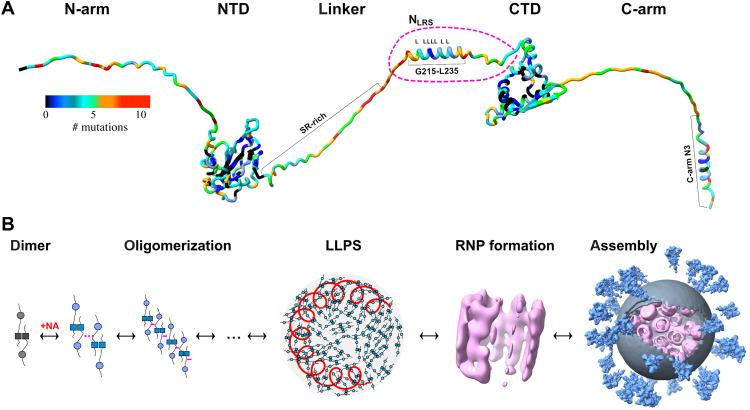
Schematics of N-protein structure and assembly. (**A**) N-protein with folded domains (NTD and CTD) and IDRs (N-arm, linker, and C-arm; all IDRs are artificially stretched for clarity). The variability of the amino acid sequence is highlighted through colors indicating for each position the number of distinct mutations contained in the GISAID genomic data base (as of July 2022) (*21*, *55*, *72*). (**B**) Current model for viral assembly: N-protein dimers, noncovalently linked in the CTD (dark squares), undergo a conformational change upon NA binding that allows oligomerization and promotes LLPS through weak protein-protein and/or protein-NA interactions. The dense phase containing genomic RNA (red coil) permits formation of RNPs [Electron Microscopy Data Bank (EMDB) 11868 (*16*)] leading to viral assembly [EMDB 30430 (*17*)].

The 46-kDa protein is composed of an N-terminal nucleic acid (NA) binding domain (NTD) and a C-terminal dimerization domain (CTD), both flanked and linked by long and flexible intrinsically disordered regions (IDRs), hereafter referred to as N-arm, linker, and C-arm (Fig. 1A) (*11*–*13*). The IDRs allow large conformational fluctuations of the N-protein dimer in solution (*14*, *15*). N-protein plays an essential structural role by scaffolding the ≈30-kb viral RNA into distinct ribonucleoprotein (RNP) particles inside the virion (*16*–*18*) in a still poorly understood assembly process. Briefly, through combination of high- and low-affinity protein-protein interactions (*12*, *13*, *19*, *20*), and in concert with several protein-NA binding sites, N-protein forms higher-order oligomers and protein-NA complexes (*20*–*24*). Ultraweak multivalent interactions promote liquid-liquid phase separation (LLPS) of N-protein and NA into droplets of biomolecular condensate (*12*, *19*, *25*–*30*). The dense phase is thought to facilitate specific interactions with the NA packaging signal leading to the generation of RNP particles (Fig. 1B) (*12*, *31*). Much of this assembly process appears to be highly dynamic, as indicated by low thermal transition temperatures, the presentation of different protein conformational changes induced by both NA binding and LLPS, and the inherent dynamic nature of the NTD and the disordered domains (*12*–*14*, *20*). Within the host cell, the assembly and packaging of RNP particles by N-protein is facilitated through interactions with viral protein NSP3, which is localized at and forms pores in the double membrane vesicles where synthesis of viral RNA occurs (*32*, *33*). In addition, N-protein also binds the endodomain of the viral M-protein to anchor RNPs to the membrane of the virion (*18*, *19*).

Cryoelectrontomography has revealed that RNPs have hexameric or tetrahedral ultrastructures (Fig. 1B) (*16*, *17*), but their molecular architectures remain unresolved. Since N-protein tightly dimerizes in the CTD and has multiple NA binding sites including the CTD and NTD, geometric considerations suggest that it may theoretically be capable of cross-linking different segments of NA chains rather than merely decorating a linear chain. However, because of the central disordered linker, N-protein exhibits a high degree of variability in the relative orientation of its folded domains (*14*, *15*). Moreover, coarse-grained molecular dynamics (MD) simulations have suggested that all domains of an N-protein dimer may interact simultaneously with a single NA chain (*15*). Thus, as-of-yet unknown protein-protein interfaces beyond the high-affinity dimer interface seem to be required for stabilizing distinct three-dimensional structures such as the RNPs. Such interfaces would present novel drug targets.

The importance of additional nucleocapsid self-association interfaces for viral assembly has long been recognized in the study of coronaviruses (*18*, *34*–*36*). For example, it was proposed that crystal packing contacts of severe acute respiratory syndrome coronavirus (SARS-CoV) N-protein CTD constitute viral assembly interfaces, which structurally matched the classical helical model for coronavirus RNPs (*34*, *36*). Other laboratories have proposed oligomerization interfaces in the C-arm (*37*–*39*), in the N-arm/NTD (*40*), or in the serine-arginine (SR)–rich region of the linker (*35*, *41*). Unfortunately, the direct experimental detection of higher-order oligomers in solution has proven difficult, partly due to unrecognized NA contamination and misinterpretation of hydrodynamic data due to the large fraction of IDRs (*20*, *26*, *42*), as well as other confounding factors such as transient intramolecular interactions between C-arm/CTD and N-arm/NTD (*12*). Nonetheless, direct evidence for very weak oligomerization of purified N-protein dimers of SARS-CoV (*43*) and SARS-CoV-2 (*20*), respectively, was obtained in analytical ultracentrifugation experiments showing dissociation equilibrium constants on the order of 1 mM.

An unexpected clue to the location of a potential new self-association interface came recently from the SARS-CoV-2 Delta variant. Its 21J clade harbors a distinguishing G215C mutation in the N-protein (N:G215C) and rose to worldwide dominance by outcompeting the initial G215-bearing 21I Delta clade without additional mutations in the viral spike protein (*21*). Studying the biophysical properties of N:G215C, we found that compared to the reference N:G215, the mutant N:G215C unexpectedly exhibits two orders of magnitude enhanced higher-order oligomerization with dimer-dimer self-association in the low micromolar range (*21*). Furthermore, it exhibits a more compact structure with higher helical content and enhanced coassembly with NA. Structurally, this mutation is adjacent to the leucine-rich sequence (LRS) 218-231 in the disordered linker. By nuclear magnetic resonance (NMR) and MD simulations, the LRS was previously shown to contain a transient helix, which was hypothesized to self-associate (*12*, *44*), and recently found to contribute to a binding interface for NSP3a (*32*). To understand the molecular origin of the enhanced dimer-dimer self-association of the N:G215C mutant, we subsequently used MD simulations that revealed how 215C keeps the helix in a more open configuration that appears better poised for potential helix-helix interactions (*21*). The relevance of this transient helix is underscored by the conspicuous lack of substitutions at this position in the mutational landscape retrieved from the Global Initiative on Sharing Avian Influenza Data (GISAID) SARS-CoV-2 genomic repository, in the midst of otherwise highly variable IDR sequences (Fig. 1A) (*21*). This protection from mutations suggests that this transient helix of the LRS may be an essential feature for viral survival.

Since the LRS is surrounded by intrinsically disordered stretches, it can be excised and its properties studied directly free from potentially confounding properties of other regions of the full-length N-protein (FL-N). In the present work, we demonstrate through biophysical studies of the LRS peptides and a series of mutants the direct linkage between helix formation and weak but cooperative assembly of coiled-coils, which constitutes a new self-association interface of N-protein that is unique among the IDRs. In corresponding experiments with the FL-N, we find coiled-coil assembly to be a driver for particle assembly and LLPS, cooperatively enhanced by NA binding through a global conformational change of N-protein. This suggests a role of LRS oligomerization in initiating viral assembly. Structure prediction and MD simulation of the LRS oligomers reveal a hydrophobic core and a pattern of hydrophobic, hydrogen bond, and salt-bridge interactions between adjacent helices, with critical positions matching amino acids highly conserved in the mutational landscape of the LRS of SARS-CoV-2. Moreover, we show that this oligomerization is conserved across related coronaviruses, in which we observe similar LRS folding–linked oligomerization in experiments with corresponding LRS peptides. Thus, the LRS helix transition and oligomerization present a new target for broadly effective, pan-coronavirus therapeutics.

## RESULTS

### Cooperative folding and assembly of LRS peptides

To study the properties of the LRS, we use a construct N:210-246 (N_LRS_). This peptide encompasses the observed transient helix (Fig. 1A) with several residues from the disordered stretches on either side to minimize end effects. The restriction to N_LRS_ provides the critical experimental advantage that its weak self-association can be studied independently at suitably high concentrations, free from CTD-driven dimerization and LLPS of FL-N. Furthermore, secondary structure changes in the LRS can be clearly assessed without overwhelming spectral contributions of the much larger remainder of the protein.

**Fig. 2.**
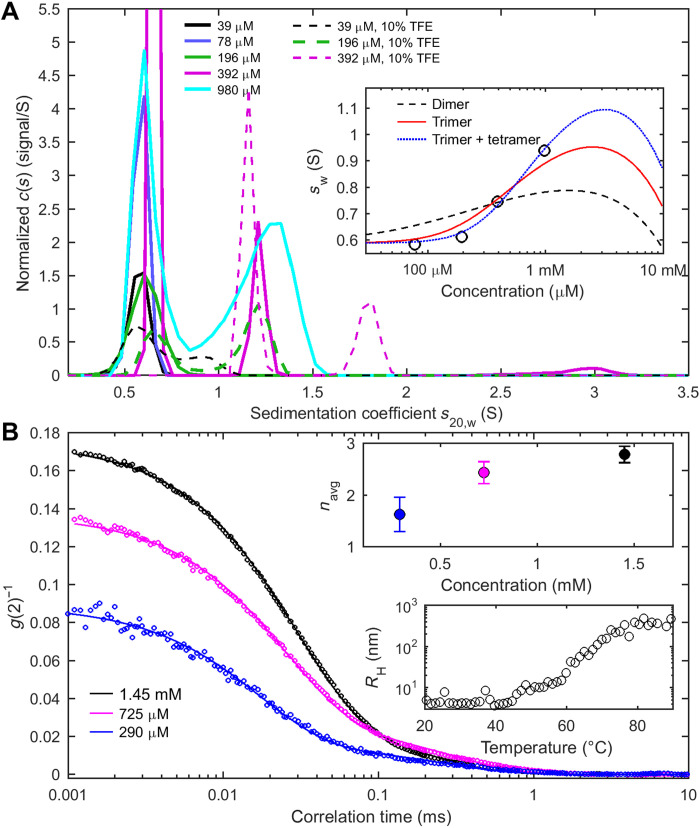
N_LRS_ peptides self-associate. (**A**) Sedimentation coefficient distributions *c*(*s*) of N_LRS_ at different concentrations in working buffer (solid lines) show monomer peaks at ≈0.6 S and trimer peaks at ≈1.3 S, the latter fraction increasing with concentration. For typical raw sedimentation boundaries, see fig. S1. For comparison, *c*(*s*) distributions in working buffer supplemented with 10% TFE are shown as dashed lines. The inset shows the weight-average sedimentation coefficients *s*_w_ (circles) with best-fit GMMA models (lines) jointly fitting the *s*_w_ and θ(222 nm) isotherms (Fig. 3A). A monomer-dimer model yields an effective *K*_D2_^*^ = 0.60 mM (black dashed line), a monomer-trimer model yields an effective *K*_D3_^*^ = 0.81 mM (red solid line), and a mixed monomer-trimer and monomer-tetramer model yields effective *K*_D3_^*^ = 1.6 mM and *K*_D4_^*^ = 1.0 mM (blue dotted line). (**B**) DLS autocorrelation data of N_LRS_ at different concentrations (circles) and best two-species fit (lines) accounting for the *z*-average peptide diffusion and traces of a ≈15 nm cluster. The top inset presents the average oligomeric state estimated by combining the peptide diffusion coefficient with the oligomer sedimentation coefficient. The bottom inset is the measured temperature dependence of the *z*-average hydrodynamic radius of N_LRS_.

First, we examine the self-association properties hydrodynamically by sedimentation velocity analytical ultracentrifugation (SV-AUC) and dynamic light scattering (DLS). These methods are based on first principles and provide gold standards for determining macromolecular size and shape in solution without interactions with matrices and not requiring labels or other modifications. The sedimentation coefficient distributions, *c*(*s*), at different N_LRS_ concentrations (Fig. 2A) exhibit a peak at 0.63 (± 0.02) S corresponding to the N_LRS_ monomer of 3.8 kDa and resolve a faster sedimenting ≈1.31 (±0.1) S population, the fraction of which increases with N_LRS_ concentration. This pattern is characteristic of the presence of oligomers in slowly reversible equilibrium with monomers (*45*). From the hydrodynamic scaling law for globular particles (*s_n_*/*s*_1_ = *n*^2/3^) the expected *s*-values for dimer, trimer, and tetramer are 1.00, 1.31, and 1.59 S, respectively. Similarly, hydrodynamic modeling of the predicted structures (see the “Molecular basis of oligomerization” section) results in theoretical *s*-values of 1.04, 1.33, and 1.69 S, respectively. Thus, the experimentally observed peak at 1.31 S is most consistent with an N_LRS_ trimer.

For corroboration, we carried out DLS experiments (Fig. 2B). DLS is highly sensitive to large particles (*46*), traces of which can be readily recognized from a τ ≈0.2 ms component of the autocorrelation function (corresponding to a hydrodynamic radius *R*_H_ ≈15 nm). Such clusters have been reported previously for FL-N and subsaturated solutions of other phase-separating systems (*20*, *47*). They are well separated from the much faster decay of the peptide signal, and accordingly, the DLS data can be fit well with a two-species model that represents both the clusters and the *z*-average diffusion coefficient of the peptide states. The latter show a concentration-dependent shift from a hydrodynamic radius *R*_H_ of 1.2 nm at 0.29 mM, where the majority of the peptides are monomeric by SV, to *R*_H_ of 1.98 nm at 1.45 mM where a significant fraction will be assembled. Combining the oligomer sedimentation coefficient with the average diffusion coefficient in the Svedberg equation yields molecular weight estimates that reflect the average assembly state, which increases from *n* = 1.6 at 0.29 mM to *n* = 2.79 at 1.45 mM (Fig. 2B, top inset). This is consistent with the gradual formation of N_LRS_ trimers within this concentration range.

**Fig. 3.**
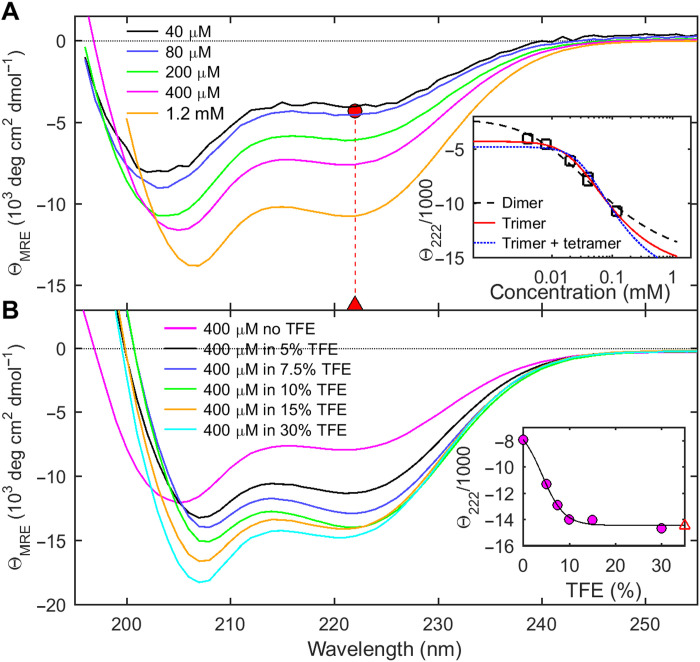
Concentration-dependent helix formation of N_LRS_. Shown are CD spectra in units of MRE values θ_MRE_, which directly reflect molecular peptide helicity. (**A**) Dependence of ellipticity on N_LRS_ concentrations. The inset shows the best fit of θ(222 nm), jointly with the *s*_w_ isotherm of Fig. 2A, with the GMMA model indicated. The GMMA monomer-trimer model results in best-fit helicities of −4290 and −16,210 deg cm^2^ dmol^−1^ for the monomer (red circle) and trimer (red triangle) state, respectively, corresponding to ≈3 helical residues in the N_LRS_ monomer increasing to ≈16 helical N_LRS_ residues per peptide in the trimer. (**B**) Solvent-induced change in helicity of 0.4 mM N_LRS_ at different concentrations of TFE. The inset shows θ(222 nm) (circles) and the best-fit two-state model (line) resulting in the best-fit ellipticity of the maximally helical state of −14,400 deg cm^2^ dmol^−1^ (red open triangle), corresponding to estimated ≈14 helical N_LRS_ residues per peptide chain.

The helicity of N_LRS_ was measured by circular dichroism (CD) spectroscopy under the same conditions, and similar concentrations as in the hydrodynamic experiments. Fig. 3A show spectra at different N_LRS_ concentrations, exhibiting minima near 208 and 222 nm typical of α helices (*48*). An overall increase in mean residue ellipticity (MRE) with concentration reveals N_LRS_ self-interactions leading to increased secondary structure. A slight shift in the first minimum from ≈203 to ≈207 nm can be discerned, while the ratio of the first to second minimum ellipticity decreases; both features also reveal disordered residues at lower concentration and increased helicity at higher concentration.

To probe the linkage between helical content and self-association in more detail, we supplemented the buffer with trifluoroethanol (TFE), a widely used cosolvent for stabilizing helical conformations (*49*). As expected, the CD spectra of N_LRS_ show a strong increase in molar helicity with TFE (Fig. 3B). Notably, the helicity of 0.4 mM N_LRS_ in 30% TFE is significantly larger than that at the highest experimental concentration of 1.2 mM N_LRS_ without TFE, suggesting that in the latter conditions the helix transition is still incomplete. This is consistent with the significant fraction of monomeric N_LRS_ observed in SV-AUC at 0.98 mM in the absence of TFE. We next performed matching SV experiments in 10% TFE. The resulting sedimentation coefficient distributions are shown as dashed lines in Fig. 2A, exhibiting larger oligomer peaks in the presence than in the absence of TFE, as well as species sedimenting at even higher *s*-values in TFE. Thus, promoting helix formation induces oligomerization and, vice versa, self-association drives helix formation, in a linked process.

**Fig. 4.**
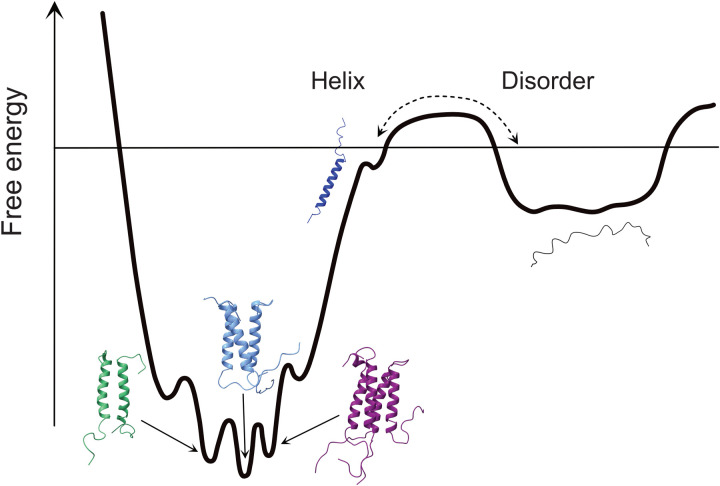
Schematics of a free energy diagram of N_LRS_ folding and oligomerization. N_LRS_ folding from the disordered state requires activation energy and is slow, while different oligomeric states of helical N_LRS_ may have similar free energy and coexist in rapid exchange.

It should be noted that from the experimental hydrodynamic data, a more complicated multistate model cannot be excluded. For example, the observed trimer could be an average of coexisting dimers, trimers, and tetramers that interconvert rapidly with each other compared to the characteristic sedimentation time—and therefore do not hydrodynamically resolve among the oligomers—but are very slow converting back to unfolded monomers. This is schematically presented in Fig. 4. Moreover, as the TFE data show, under conditions both promoting folding and self-association even higher oligomers can form.

The energetics of the folding-linked oligomerization is reflected in both the isotherms of weight-average sedimentation coefficients and MRE as a function of N_LRS_ concentration (insets in Figs. 2A and 3A). Focusing on conditions in the absence of TFE, we applied global multimethod analysis (GMMA) to probe their consistency and to derive binding constants. While a monomer-dimer model fails to describe the data (Fig. 2A), the cooperative monomer-trimer model fits both isotherms well with a *K*_D_^*^ of 0.81 mM (where monomer and oligomer are equimolar; total free energy of trimerization Δ*G*_trimer_ = −8.4 kcal/mol), with a slight improvement allowing for coexisting trimers and tetramers. Since GMMA decomposes the experimentally measured ellipticity into contributions from monomer and trimer species, it is possible to quantify the extent of folding in each state. This results in an estimated three helical residues in the monomer, increasing to a helix of 16 residues length in each chain of the trimer. This may be compared with the maximal peptide helicity derived from fitting a two-state model to the TFE dependence of ellipticity (Fig. 3B), which results in a similar estimate of 14 helical residues per N_LRS_ chain in the folded state in TFE.

Considering that other IDR peptides of N-protein were previously proposed to contain transient helices potentially linked to protein-protein interfaces, the question arises whether the folding-linked self-association is unique to the LRS. To this end, we carried out analogous SV and CD experiments of peptides of the N-arm (N:1-43), the SR-rich region of the linker (N:181-209), the C-arm (N:365-419), and its C-terminal N3 region (N:390-419) (*50*). Notably different from the N_LRS_ peptide, all sediment at *s*-values expected for their monomeric state without signs of self- association, and all peptides showed concentration-independent secondary structure (fig. S2). Moreover, all peptides exhibited CD spectra characteristic of disorder and little helicity at 222 nm [θ_MRE_(222 nm) values in 10^3^ deg cm^2^ dmol^−1^ are −1.1 for N-arm, −2.1 for SR-linker, −1.2 for C-arm, and − 3.3 for C-arm-N3, compared to −10.7 for N_LRS_].

To probe whether helicity could be induced in the other peptides if a driving force is applied, CD experiments were also carried out in 30% TFE. Still none of the peptides showed any concentration-dependent secondary structure under these conditions, and a distinct helical signature appears only for the C-arm peptides [θ_MRE_(222 nm) values in TFE are −5.3 for C-arm and − 12.9 for C-arm-N3, compared to −15.4 for 0.4 mM N_LRS_] (fig. S2). The helicity in the C-arm peptide can be entirely attributed to its C-terminal N3 region, which coincides with a predicted transient C-terminal helix (Fig. 1A). However, on the basis of the present experiments, folding of this helix is significantly weaker than that of N_LRS_ and is not linked to concentration-dependent self-association—this is unique to N_LRS_.

### Molecular basis of oligomerization

**Fig. 5.**
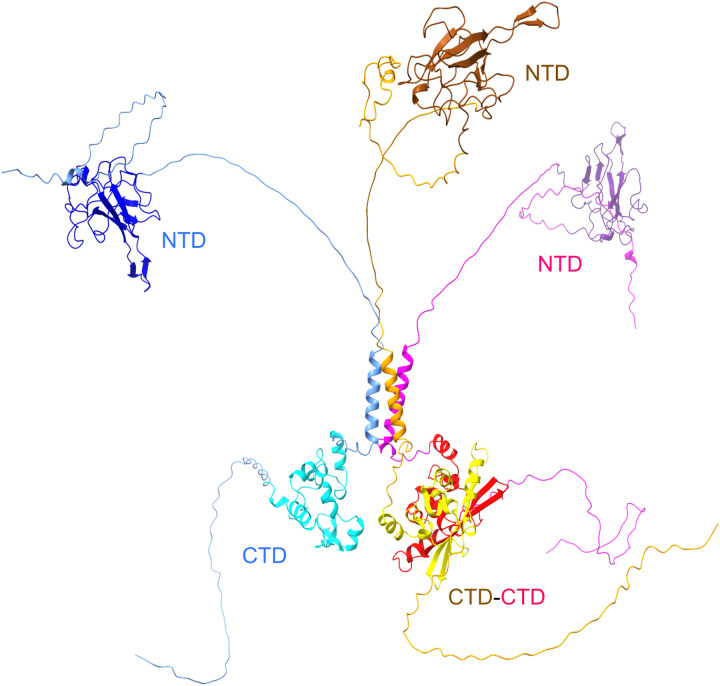
Configuration of a trimeric FL–N-protein predicted by ColabFold. Three FL-N sequences are predicted to simultaneously have CTD dimer contacts and LRS trimer contacts. The disordered segments are not well predicted and are in quasi-random configuration; therefore, the linker IDR 180-210 and the C-arm are artificially stretched for clarity.

Since N-protein has a high-affinity dimerization interface in the CTD not far from the LRS, the question arises how simultaneous dimerization in the CTD and trimerization in the LRS is sterically possible in the FL-N and what structural arrangements might be required to accommodate them. To address this question, we took advantage of ColabFold (*51*), a recent extension of AlphaFold2 for the prediction of protein complexes (*52*, *53*). Since it is not tailored to the prediction of disordered states, because the training set contains crystallographic structures, it will have a strong bias to predict the most folded structures possible and provide no confidence in the predicted disordered regions. Nonetheless, disordered regions will, at a minimum, be continuous and nonoverlapping chains that will present one out of a continuum of possible configurations. Figure 5 shows a structure prediction of three FL-N with the helical LRS arranged a trimeric structure and two chains dimerizing through CTD contacts. The high flexibility of the disordered regions ensures the absence of steric clashes while oligomerization interfaces can engage. Other higher-order architectures are conceivable and predicted by ColabFold, such as a tetramer arranged as two CTD-linked dimers joined in a tetrameric LRS and a hexamer connected in three dimer CTD interfaces and two trimeric LRS helices (fig. S3).

Focusing on the nature of the LRS oligomers, we generated ColabFold predictions for multimers of the N_LRS_ peptide, resulting in coiled-coils with a high degree of symmetry (fig. S4). Overall, good confidence scores were achieved, highest for the trimer. The complementary residues predicted to make interhelix contacts coincide with residues that are highly conserved among related coronaviruses and show an extremely low incidence of mutations in the SARS-CoV-2 genomic repository, including L219, L221, L222, L224, R226, E231, and K233 (fig. S4).

**Fig. 6.**
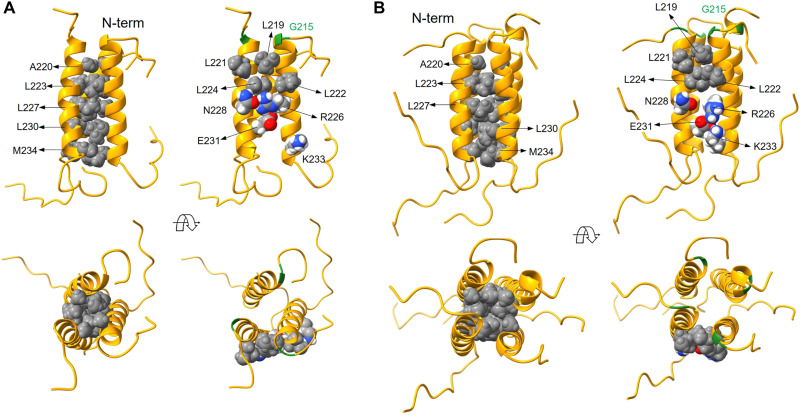
MD simulations highlight residues that stabilize N_LRS_ oligomers. Shown are snapshots from the MD simulations for the trimer (**A**) and tetramer (**B**). The cores of the oligomers are formed by five nonpolar residues in a well-packed configuration (left column of each panel). More specific interactions stabilizing the complex surfaces are (right column of each panel): a hydrophobic surface cluster formed by four leucine residues, two salt bridges (R226-E231 and K233-E231), and an H-bond interaction between polar (N228) and charged (R226) residues. Helix backbones drawn as gold ribbons; interacting residues rendered as atom-based Van der Waals spheres (nonpolar residues in gray; polar and charged residues in color: white, H; red, O; gray, C; blue, N). G215 is shown in green for reference.

MD simulations of the oligomers at near experimental conditions show significant adaptation of the interfaces and stronger interactions between the critical residues that stabilize the complex surfaces (Fig. 6). Hydrophobic residues that likely initiate the complexation process are clustered on one side of the helix and become more compact in the complex, rendering the interactions in the core formed by L220, L223, L227, L230, and M234 strictly nonpolar due to the complete exclusion of water (Fig. 6). At the same time, solvent-exposed residues L219 and L222 in one helix and L221 and L224 in an adjacent helix form an extended hydrophobic patch that stabilizes the complex surface in all the oligomers (Fig. 6). In addition, R226 in one helix forms a salt bridge with E231 in the adjacent helix, further stabilizing the complexes; formation of transient N228-R226 H-bond interactions is also observed depending on the complex.

Snapshots along the entire 30-ns simulation for monomers and oligomers are shown in fig. S5. While there was no interconversion of oligomeric states observed on the time scale of the simulations, for qualitative comparative analysis of the stability of different oligomers, it is interesting to examine the frequency of a given residue interaction throughout the dynamics trajectory as a proxy for its relative strength (fig. S6). The R226-E231 salt bridge is persistent—presumably stronger—for the trimer, whereas in the tetramer its frequency decreases in favor of E226-K233. The four-leucine patch on the surface appears to be more compact and persistent in the tetramer. However, electrostatics and H-bond interactions between side chains in solvent-accessible regions of proteins are typically stronger than hydrophobic interactions (*54*). This analysis, and the fact that all the oligomers are stable throughout the simulations, supports the mechanistic view depicted in Fig. 4 of a mixed oligomeric state, with a higher population of trimers coexisting with lower populations of dimers and tetramers. Calculations of CD spectra from the dynamics trajectory show that all the oligomers exhibit stronger helicity than the monomer. The estimated time average numbers of helical residues per chain are 19 for the monomer and 21 in the trimer. The increase of oligomeric helicity is qualitatively consistent with the oligomerization-linked folding observed in CD.

To further examine the role of individual residues in helix formation and oligomer stabilization, we explored several mutants in MD simulations and experiments probing their oligomeric state, helicity, and stability. Results are described in detail in figs. S7 and S8 and briefly summarized in the following. In a first set, mutations disrupt oligomerization at key residues: (i) N_LRS_:L222P introduces a hinge disrupting the cohesiveness of the surface hydrophobic patch; (ii) N_LRS_:R226P creates a less acute hinge but eliminates the salt bridge to E231; (iii) a group of three mutations of L224—a position critical for the surface hydrophobic patch—weaken hydrophobic forces between adjacent helices through substitution by either a hydrophobic residue of smaller size (N_LRS_:L224A), a polar residue (N_LRS_:L224S), or a charged residue (N_LRS_:L224D); (iv) contributions of L219 to the hydrophobic surface cluster are abrogated by alanine substitution (N_LRS_:L219A). In CD experiments, all six peptide mutants show drastically reduced helicity compared to N_LRS_, while in SV experiments, no oligomerization can be detected for any of these mutants (fig. S7).

In a second set, mutants were created to enhance oligomerization. These are based on the previous observation of enhanced assembly properties of FL-N:G215C in vitro, in a study motivated by the conspicuous role of the G215C mutation in the SARS-CoV-2 Delta variant (*21*). In that work, MD simulations of the monomeric N_LRS_:G215C peptide clarified the role of C215 in repositioning the N-terminal disordered residues adjacent to the helix upward to improve its accessibility for self-association. In the present work, the previous results are corroborated in MD simulations of the oligomers and augmented by experimental studies with the N_LRS_:G215C peptide. In SV and CD, it displays both enhanced self-association and helicity (fig. S8), mimicking the behavior of the full-length construct, N:G215C. Slightly enhanced self-association under nonreducing compared to reducing conditions demonstrates contributions of disulfide bonds to the assembly. To eliminate the potential for disulfide bonds, we also studied the analogous peptide N_LRS_:G215S. Its properties in concentration-dependent helix formation by CD and oligomerization by SV are qualitatively similar to G215C in reducing conditions and indistinguishable in MD simulations (fig. S8). In summary, at similar concentrations, all of the mutants of this second set exhibit stronger self-association than the reference peptide N_LRS_. Assuming the mechanism of self-association and linked folding to be identical, from the similarity of CD spectra and SV profiles of 0.4 mM N_LRS_:G215C and 1.2 mM N_LRS_, it is possible to estimate the free energy enhancement to be ≈−1.3 kcal/mol. The enhancement of N_LRS_:G215S is slightly larger. Similar to N_LRS_ in TFE, the stronger driving force to self-assemble can populate higher oligomers at high concentrations.

The enhancing and abrogating mutants allow us to compare the concentration-dependent stability of the N_LRS_ coiled-coil structure through temperature-dependent CD experiments (fig. S9). For the reference peptide N_LRS_, a transition occurs at high temperature to a state that is still largely helical but exhibiting an increased disordered component as judged by the shift of the first minimum at ≈208 nm to lower wavelengths. A distinct concentration dependence of the transition (from 42°C at 0.4 mM to 52.5°C at 1.2 mM) demonstrates the stabilizing effect of oligomerization. The transition temperatures approximately coincide with the onset of particle formation observed in DLS (Fig. 2B). As discussed above, the destabilizing mutant N_LRS_:L222P shows little helicity at room temperature but increases helicity by approximately twofold in a first transition at 36.5°C (presumably strengthening hydrophobic interactions in the surface cluster can overcome the destabilization by P222), before unfolding into a largely disordered state at 42.5°C. The stabilizing mutant N_LRS_:G215C shows the opposite behavior: Similar to N_LRS_, a single transition occurs but at a higher temperature of 55.8°C (at 1.2 mM), and it assumes a slightly lower unfolded fraction in the high-temperature state than N_LRS_ (fig. S9).

In summary, the N_LRS_ oligomers feature a hydrophobic cluster and electrostatic interactions at key residues, as derived from MD simulations, consistent with the initial ColabFold predictions, and corroborated experimentally by assembly-promoting and abrogating point mutants.

### The mutational landscape of the LRS from a structure/function viewpoint

**Fig. 7.**
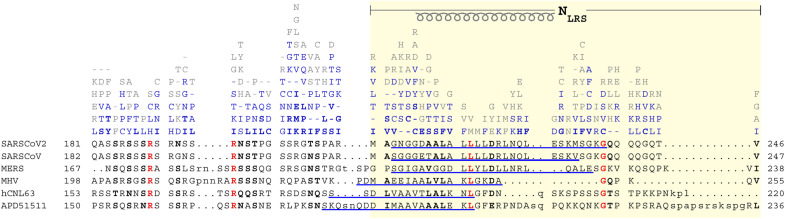
LRS is conserved in both the mutational landscape of SARS-CoV-2 linker IDR and in alignment with related coronaviruses. The linker region 181-246 of the Wuhan-Hu-1 SARS-CoV-2 (P0DTC9.1) N-protein is aligned with SARS-CoV (P59595.1), MERS (YP_009047211.1), murine hepatitis virus (NP_045302.1), human coronavirus NL63 (Q6Q1R8.1), and the 229E-related bat coronavirus APD51511.1. Conserved identical residues are highlighted in bold red, and conserved similar residues are highlighted in bold black. Above the aligned sequences are observed distinct mutations of SARS-CoV-2 observed among 3.83 million high-quality sequences uploaded to GISAID between January 2020 and August 2022. The mutations are ordered and colored by number of genomes found to contain that specific mutation: 10 to 100 (light gray), 100 to 1000 (light blue), and >1000 (bold blue). The underlined sequences correspond to trimeric helices predicted by ColabFold.

The detailed molecular model of the LRS provides a framework for interpreting viable mutations found in the GISAID repository of SARS-CoV-2 genomes (*55*). Figure 7 shows the sequence alignment of the linker region of SARS-CoV-2 and related coronaviruses. It also shows for each position the variants found in 3.83 million entries of the repository, sorted by relative incidence. As described previously, the disordered linker region is richly mutated—with an average of 5.3, and up to 11, distinct amino acid substitutions at each residue—with the exception of the helical region in the LRS (Figs. 1A and 7) (*21*). The lack of deposited SARS-CoV-2 genomes containing mutations in the range of 221 to 227 and at positions 231 and 233 suggests that these residues are essential for viral survival. This matches most of the lysine residues contributing to the nonpolar core, the hydrophobic surface cluster, and the salt bridge.

Nonetheless, the exclusion of mutations is not complete, and in the context of the structural model developed here, most of these rare mutations can be rationalized. For example, the arginine R226 forming the salt bridge with E231 is, at very low incidence (0.0053%, *n* = 202 of 3.83 million genomes), found to be replaced by lysine, which has a similarly long acidic side chain. However, the substitution of R226 with isoleucine at even lower incidence (*n* = 41) is unclear. The glutamic acid E231 is rarely replaced, in turn, with aspartic acid (*n* = 337), which is likely to engage in similar electrostatic interactions. The lysine K233 is found to be replaced in even fewer cases with asparagine and arginine (*n* = 96 and *n* = 26, respectively), which may partially retain electrostatic interactions or H-bonds with R226. Furthermore, statistics over the MD trajectory show that while the R226-E231 salt bridge is persistent for the trimer, it may be transiently replaced by E226-K233 favoring a tetrameric state.

Examining the seven leucine residues from 219 to 230, it can be discerned that these are replaced rarely and exclusively by phenylalanine, isoleucine, valine, or methionine. Presumably, by virtue of their hydrophobic nature, these substitutions do not severely disrupt the hydrophobic interaction networks (based on the MD simulations of mutations of L224 above). This may explain why the most common replacement is phenylalanine (*n* = 1335 at position 219, *n* = 928 at 221, *n* = 474 at 224, and *n* = 5745 at 230), which is large enough to keep the surrounding hydrophobic residues close together in a compact configuration.

One of the most conserved residues in the LRS of SARS-CoV-2 is L222; conspicuously, it is also completely conserved across related coronaviruses (below). Nonetheless, a mutation L222M is found in *n* = 79 viable SARS-CoV-2 genomes. To examine this substitution, we generated the methionine substituted peptide N_LRS_:L222M and studied it by MD, SV, and CD. In MD simulations, methionine is not as effective as a branched leucine in stabilizing the compact hydrophobic surface patch, but it is the only residue long and flexible enough to bend upward and interact hydrophobically with the disordered N-terminal segments (fig. S10A). In the trimer, these interactions also occur with adjacent helices, a feature not observed in any other cases. Thus, the partial loss of hydrophobic surface stabilization is partially compensated by a gain of hydrophobic interaction with the N-term. Consistent with this idea, CD and SV experiments show concentration-dependent folding and oligomerization of N_LRS_:L222M that is similar to the reference N_LRS_. In a CD temperature scan, the phase transition is at 46.4°C at 1.2 mM, which is between the values corresponding to N_LRS_ at 0.4 and 1.2 mM (fig. S10, B to D). Since the naturally occurrences of L222M mutations took place in the Delta variant, we also studied a double mutant N_LRS_:L222M/G215C in SV and observe similar enhancement as the N_LRS_:G215C peptide. Thus, even the highly conserved residue L222 can be replaced, at some fitness cost, by an amino acid that largely maintains the assembly-linked folding function.

### The role of LRS self-association in viral assembly

The study of N_LRS_ and other IDR peptides above sheds light on the role of the LRS in the context of the FL protein and its higher-order assembly. Without any mutations, we saw N_LRS_ self-associates weakly with ≈mM affinity, similar to the higher-order self-association described previously with less detail for FL-N dimers (*20*, *43*). Also, we have studied in-depth the naturally occurring mutation G215C that strongly enhances self-association in both the N_LRS_ peptide and the FL protein (*21*). We find similar enhancement with N_LRS_:G215S, and by virtue of the oxygen in serine replacing the sulfur of cysteine, this rules out any potential involvement of disulfide bonds and removes any ambiguity of the key role of LRS in the enhanced self-association by 215C.

**Fig. 8.**
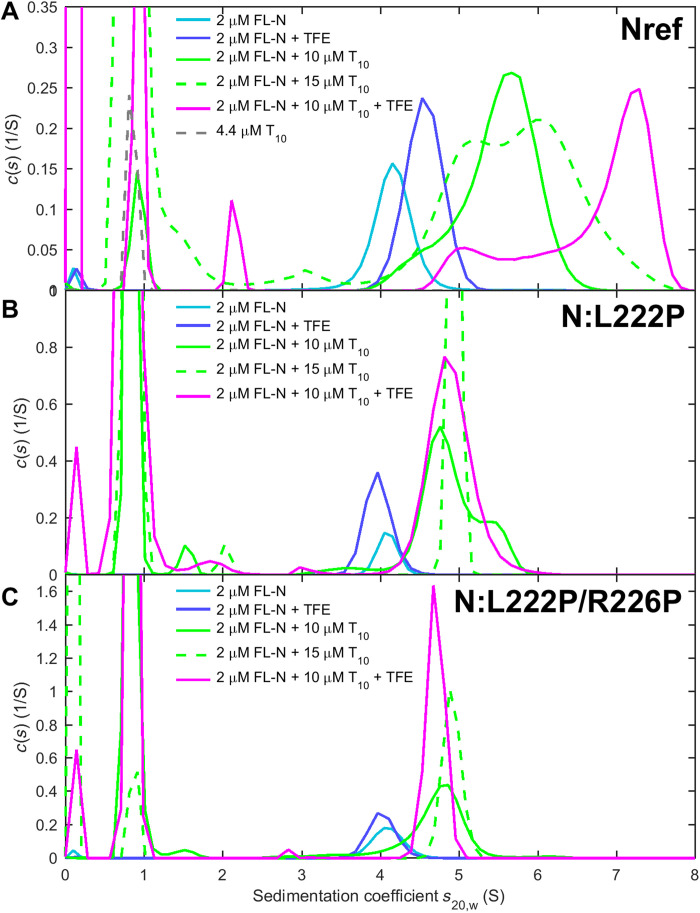
Self-association of FL-N and NA-liganded FL-N is mediated by LRS assembly. SV experiments are carried out with three FL-N protein constructs (cyan): (**A**) the ancestral reference N-protein, (**B**) the mutant N:L222P that diminishes LRS folding and assembly, and (**C**) the double mutant N:L222P/R226P abolishing LRS assembly. Sedimentation coefficient distributions are shown in the presence of 10% TFE (blue), an excess of oligonucleotides T_10_ (green), and both 10% TFE and T_10_ (magenta).

Another avenue to enhance self-association emerging from the study of LRS peptides is the stabilization of transient helices in TFE. To test this, we studied FL-N in the presence of TFE. As expected, by CD, we observe a significant increase in helicity (fig. S11), and in SV experiments, we observe a concomitant significant increase in the peak sedimentation coefficient (Fig. 8A). Thus, FL-N mimics the enhanced self-association in TFE. We note that among all IDR regions of N-protein, enhanced self-association induced by TFE was exclusive for N_LRS_.

The peptide studies also led us to the proline mutants N_LRS_:L222P and N_LRS_:R226P with strongly reduced self-association and helicity. To test their effect in the context of the FL-N, we expressed FL-N:L222P and the double mutant FL-N:L222P/R226P. As shown in Fig. 8 (B and C), FL-N:L222P opposes the enhancement of self-association by 10% TFE, and FL-N:L222P/R226P completely abolishes the TFE effect. This corroborates that in the context of the FL protein, TFE acts on the LRS as in the peptide and that the mutants exert the same enhancement or disruption as in the peptide. Thus, the LRS in the FL protein functions similarly and independently as the excised LRS peptide.

N-protein plays a central role in viral assembly, which is an orchestrated process initiated by NA binding. In previous work with FL-N (*20*), we have shown that binding of a short oligonucleotide causes a conformational change in FL-N that is associated with increased helicity and higher-order self-association, a mechanism hypothesized to constitute a first step in assembly. We have more recently shown that this self-association in the presence of oligonucleotides is strongly enhanced in the FL-N:G215C mutant, which implicates the LRS as the site of the protein interaction interface (*21*). In the present study, to examine further the interplay between NA binding and N-protein conformation and oligomerization, we can take advantage of the enhancing conditions of TFE and the abrogating proline mutants to modulate LRS self-association. An initial control experiment by SV shows the absence of binding of T_20_ to the N_LRS_ peptide (fig. S12), consistent with the lack of significant net charges in the LRS. For studying the NA binding to FL-N, we use an excess of oligonucleotide T_10_ that binds FL-N with high affinity. In contrast to longer oligonucleotides, T_10_ cannot simultaneously bind multiple NA binding sites on FL-N (*20*). As it does not support multivalent binding or scaffolding, ligation by T_10_ reveals ligand-induced protein-protein interactions between FL-N dimers (Fig. 8A) (*20*). These can be studied directly through observation of the time average sedimentation coefficient in SV. As may be discerned from the shift of the sedimentation coefficient distributions of FL-N/NA to higher *s*-values in Fig. 8A (magenta compared to green curve), TFE magnifies the self-association of FL-N when liganded by T_10_, significantly exceeding the TFE-induced enhancement in the absence of NA. By contrast, both FL-N:L222P and FL-N:L222P/R226P show little NA-induced self-association neither with nor without TFE (Fig. 8, B and C). This allows us to identify the LRS as the protein interaction interface for higher-order self-association when FL-N is liganded by NA. This can naturally explain the previously observed increase in helicity upon NA binding (*20*).

The residual shift in *s*-value of the NA-liganded compared to the unliganded protein in Fig. 8C (in the 4 to 5 S range) reflects the effect of bound T_10_. Since the double mutant is free from confounding effects of self-association, the increased sedimentation rate can be interpreted with simple hydrodynamic models. The ratio of measured peak *s*-values indicates that T_10_-liganded N-protein sediments 17% faster although bound T_10_ increases the mass of the particle by only 6.3%. This unequivocally demonstrates compaction of N-protein, corresponding to a decrease of the hydrodynamic radius from 5.4 nm in the absence to 4.9 nm in the presence of T_10_. Thus, the impact of the NA binding on the LRS is mediated by long-range conformational changes.

**Fig. 9.**
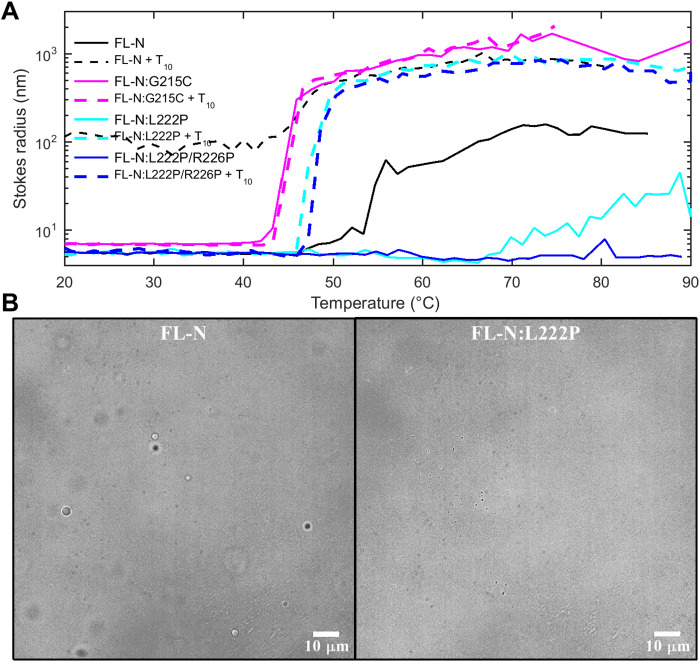
LRS mutants modulate particle formation and LLPS of FL-N. (**A**) The onset of particle formation observed by DLS. *Z*-average hydrodynamic radii as a function of temperature are shown for 3 μM ancestral FL-N (black), the LRS self-association enhancing mutant FL-N:G215C (magenta), and the abrogating mutants FL-N:L222P (cyan) and FL-N:L222P/R226P (blue), all in the absence (solid lines) and presence of 10 μM NA ligand T_10_ (dashed lines). (**B**) LLPS was studied by optical microscopy using 5 μM samples of ancestral FL-N (left) or the LRS self-association suppressing mutant FL-N:L222P in the presence of 10 μM T_10_ in 10.1 mM Na_2_PO_4_, 1.8 mM KH_2_PO_4_, 2.7 mM KCl, 10 mM NaCl, and 10% polyethylene glycol (pH 7.40) at room temperature. Images shown were taken after 6 hours.

A second function of N-protein in viral assembly is the formation of macromolecular condensates that are thought to promote scaffolding of viral RNA into RNPs (Fig. 1B). LLPS of N-protein is enhanced at higher temperature (*20*, *25*), and therefore, temperature-dependent DLS can be used to observe the onset of particle formation (Fig. 9A). The LRS assembly–promoting mutation FL-N:G215C lowers the transition temperature and increases the average particle size. By contrast, the mutation FL-N:L222P that suppresses LRS assembly strongly inhibits particle formation, and the abrogating mutant FL-N:L222P/R226P completely abolishes it. The ligation of N-protein by T_10_ restores particle formation for both FL-N:L222P and FL-N:L222P/R226P, although with a higher transition temperature. Evidently, the additional driving force for LRS assembly provided by NA binding is sufficient to favor particle formation at higher temperature (Fig. 9A), but not at room temperature (Fig. 8, B and C), consistent with the substantially hydrophobic nature of the LRS interactions. At a larger scale, particles from LLPS-driven condensate formation can be observed through microscopy. As may be discerned from Fig. 9B, under conditions where FL-N forms condensate droplets, none could be observed for FL-N:L222P.

### Preservation of LRS oligomerization across coronaviruses

We have examined above the structural basis for the transient folding-linked oligomerization of the LRS, which contributes both to the early assembly steps with NA and LLPS to create molecular condensates. We have also found evidence in the mutational landscape of SARS-CoV-2 genomes that mutations interfering with the linked folding/assembly function are lethal, and therefore, the LRS is highly conserved in all SARS-CoV-2 genomes. As may be discerned from the sequence alignment in Fig. 7, this conservation also extends to key residues, such as L222, across the related betacoronaviruses SARS-CoV (P59595.1), MERS (YP_009047211.1), murine hepatitis virus (MHV) (NP_045302.1), the common cold alphacoronaviruses NL63 (Q6Q1R8.1), and the 229E-related bat coronavirus APD51511.1. This poses the question whether the analogous regions in the related coronaviruses might facilitate similar folding-linked oligomerization processes.

**Fig. 10.**
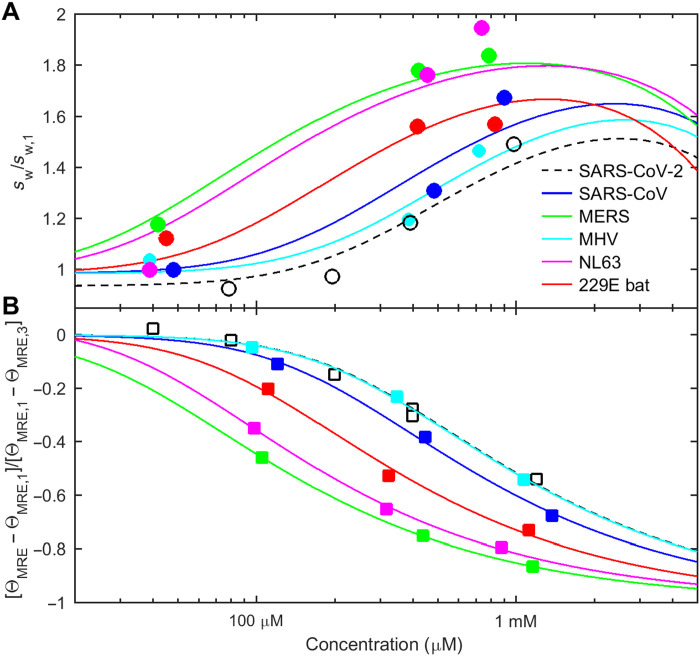
Related coronaviruses exhibit similar concentration-dependent self-association and linked folding of their LRS. Peptides aligning with the LRS of SARS-CoV-2 comprising oligomeric helices predicted in ColabFold were created for SARS-CoV, MERS, MHV, human coronavirus NL63, and the 229E-related bat coronavirus APD51511.1. (**A**) From SV experiments, the weight-average sedimentation coefficient as a function of concentration (symbols), normalized relative to the monomer *s*-value measured at low concentrations, increases with concentration. (**B**) From CD experiments, the measured MRE at 222 nm decreases at higher concentration indicating increased folding. For clarity, the symbols show θ_MRE_ scaled as fractional change. In both panels, the solid lines depict the global best GMMA fit of a monomer-trimer self-association model to both isotherms of each LRS peptide simultaneously. Best-fit binding parameters are listed in table S1.

Application of ColabFold predicts helices that can form trimeric structures for each of the coronaviruses LRS regions. Helical residues are underlined in Fig. 7, and structures are in panel A of figs. S13 to S17. Because of the predicted slight shift in the helix positions, we created LRS peptides for all coronaviruses that were shifted accordingly so as to comprise the entire predicted helices plus 5 to 10 additional residues on either side (table S1). Similar to the SARS-CoV-2 LRS, sedimentation coefficient distributions from SV experiments show that these coronavirus LRS peptides are largely monomeric at ≈100 μM and below, but at ≈1 mM, the majority of chains are oligomeric consistent with a trimeric state (panel B of figs. S13 to S17). Moreover, all species exhibit a concentration-dependent CD spectrum, with significantly enhanced helicity and reduced disorder at high concentrations (panel C of figs. S13 to S17). (Also, peptides comprising strictly only the region aligning with 210 to 246 of SARS-CoV-2, as shown with yellow highlight in Fig. 7, yield similar experimental results, except for the shortest peptide from MHV, which, in this more truncated version, displayed larger clusters instead of oligomers.) Quantitatively, the isotherms of weight-average sedimentation coefficients and fractional folding of each peptide can be described as a reversible unfolded monomer/folded trimer transition (Fig. 10). The best-fit trimerization affinity of SARS-CoV and MHV is very similar to that of SARS-CoV-2 (with effective *K*_D_^*^ = 0.5 to 0.81 mM), while MERS, NL63, and bat 229E exhibited stronger oligomerization (*K*_D_^*^ = 0.11 to 0.29 mM) (table S1), although the formation of tetramers and higher oligomers may be indicated for some by the steep concentration dependence of assembly (Fig. 10).

## DISCUSSION

Our data show that SARS-CoV-2 N-protein has a second protein-protein interaction interface besides the well-known high-affinity dimerization site in the CTD. The new site is located in the LRS of the intrinsically disordered linker and has been previously obscured by the transient nature of the interacting helices and by the relative weakness of the interaction. Nonetheless, precisely because of the dynamic nature of the interaction, it can permit additional functions that require soluble N-protein, play its crucial role in promoting LLPS as opposed to the growth of amorphous or fibrillar aggregates (*56*), and, at high concentrations—such as within condensate droplets—contribute to shaping protein-NA assemblies. In this regard, although the detailed structure and assembly of RNPs are still unclear, entropic colocalization effects generated by high-affinity CTD interactions in concert with N-protein scaffolding on NA can be envisioned to effectively strengthen LRS-LRS interactions to create stable three-dimensional structures.

N-protein is highly multifunctional and highly expressed in infected cells. It has been estimated to amount to ≈1% of total cellular protein (*5*, *57*), which in a back-of-the-envelope estimate corresponds to 10 to 100 μM N-protein. This is below the effective *K*_D_ measured for the LRS-LRS interactions. This ensures N-protein is still soluble and available for simultaneous noncompetitive engagement of N-protein in multiple cellular processes. However, at the same time, it renders N-protein poised for phase separation and assembly with little increase in local concentration.

Our finding that the LRS is critical for LLPS is consistent with recent work by Lu and colleagues (*19*) identifying the L/Q rich region key for phase separation. It is also consistent with observations from cross-linking mass spectrometry experiments where cross-links of lysine residues adjacent to the LRS helix are enriched in condensates (*19*, *29*). The data presented here further clarify the locus and mechanism of this essential N-protein function in LLPS as a result of transient folding-linked oligomerization. Weakly interacting coiled-coils represent “stickers” with weak multivalent interactions characteristic of phase-separating proteins.

On the basis of SV and MD data of the LRS peptide, the most likely oligomeric state is the trimer, but tetramers cannot be excluded, and under conditions of strong assembly, we have observed even higher oligomers. Considering the weak interaction, it is conceivable that the LRS has a polymorphic ability to assume different oligomeric states in different complexes. Structural polymorphism of coiled-coils has been well documented (*58*), and polymorphism was also found in the coiled-coil oligomers in the stalk domain of the SARS-CoV-2 spike protein (*59*). Polymorphism of N-protein may be helpful to potentially stabilize different cross-links in the RNP structure. Moreover, the question of the stoichiometry and strength of LRS-LRS interactions is highly relevant for LLPS, where these factors affect the phase behavior of the protein and size distribution of the resulting droplets (*60*, *61*). A flat energy landscape allowing for the transition between different LRS oligomeric states (Fig. 4) may also have contributed to the observed variability in detailed phase separation conditions (*62*).

N-protein is highly flexible, with the disordered linker permitting the LRS to make interchain contacts along nearly the entire N-protein (*14*), and this flexibility also bears the potential of functioning as a conformational switch. For example, a global conformational change leading to compaction was observed upon binding of a single LRS helix to NSP3a, an interaction that was found to be competitive with NA binding (*32*). Binding of NA (in the absence of NSP3a) appears to induce a different global conformational change in N-protein: In an NMR study, Pontoriero and colleagues (*23*) observed that binding of RNA to the NTD perturbs resonances in the LRS, and, measuring cross-talk between LRS and NTD in the absence of NA, they proposed intramolecular interactions to provide a regulatory mechanism for macromolecular interactions. Similar observations were made with a heparin ligand for the NTD (*63*). Unfortunately, the molecular details of this conformational change are still unclear. At a minimum, it would be expected that charge neutralization accompanying polyanion binding to the NTD alters the global conformation of FL-N. In the present work, we found that although oligonucleotides do not bind to the LRS, once bound to FL-N (most likely at the high-affinity NA binding site in the NTD), they cause compaction of FL-N and significantly enhanced LRS folding and self-association (Fig. 8). This cooperativity between NA binding and N-protein self-association at the LRS may act as a switch to initiate RNP assembly while enabling other N-protein functions in the absence of NA.

Other regions of FL-N also contribute to RNP assembly in concert with LRS, as elegantly shown by Carlson *et al.* (*24*). Despite their weaker helix propensity and absence of self-association observed here, they may become significant, for example, under conditions where different copies of N-proteins are already colocalized in an RNP or bound to an NA scaffold. Redundancy in protein-protein contacts, like polymorphism, may be useful to accommodate different structural elements of genomic RNA into the RNP. Similarly, studies of deletion N-protein constructs show that the LRS strongly favors LLPS but is not exclusively required in the presence of RNA (*19*). In particular, the C-arm, which we found can also assume a helical state in the N3 region, has been implicated in LLPS and RNP formation (*24*, *26*, *29*).

Although SARS-CoV-2 N-protein has a high sequence similarity to other betacoronaviruses, it exhibits high sequence diversity in circulating virions (*57*). Analysis of the N-protein genomes in the GISAID repository shows that, on average, each position can be occupied by more than four different amino acids, significantly exceeding the variability of all other structural SARS-CoV-2 proteins, including the spike protein (*21*). In light of this, the importance of the LRS-LRS interaction is underscored by the extraordinarily high sequence conservation of the LRS in the mutational landscape of viable SARS-CoV-2 genomes, which is nearly complete after allowing for rare substitutions that can functionally preserve the LRS self-assembly. Moreover, as we have shown experimentally, the folding-induced self-association motif of the LRS is conserved across a range of related coronaviruses, emphasizing its crucial role in viral assembly. As mentioned above, this notion matches the enhanced infectivity of the 21J clade of Delta SARS-CoV-2, which has as a distinguishing feature the G215C mutation causing enhanced LRS self-association (*21*). In addition, other N-protein functions may also depend on the ability of the LRS to assume a helical state and contribute to the lethality of LRS mutations, such as the interaction of LRS with NSP3a (*32*). Because of the high degree of sequence conservation, the N-protein LRS has been proposed as a peptide for vaccine development (*64*). Targeting the LRS to prevent N-protein self-association may open a new avenue for the development of pan-coronavirus therapeutics.

## MATERIALS AND METHODS

### Experimental design

Goal of the experiments was to examine the role of the LRS of SARS-CoV-2 N-protein. To this end, the LRS peptide was first studied separately in biophysical experiments reporting on the oligomeric state and protein-protein interactions in first-principles–based hydrodynamic techniques and probing their secondary structure using CD spectroscopy. These experiments were complemented by structure predictions and MD simulations. The resulting hypotheses on key residues were tested experimentally in mutant peptides and compared with the mutational landscape. Last, FL–N-protein was studied, first showing experimentally that the LRS functions equivalently in the FL-N as in the peptide and then taking advantage of analogous mutants to probe the impact of enhanced or disrupted LRS oligomerization on N protein-protein interactions, protein-NA interactions, particle formation, and LLPS.

### Proteins, peptides, and oligonucleotides

SARS-CoV-2 N-protein accession #YP_009724397 was acquired from EXONBIO (San Diego, CA, catalog no. 19CoV-N150). For LLPS imaging experiments, FL-N with and without the L222P mutation was also expressed and purified as described in detail previously (*21*). Briefly, the protein including 6His with Tobacco Etch Virus (TEV) cleavage site was cloned into the pET-29a(+) expression vector and transformed into One Shot BL21(DE3)pLysS *Escherichia coli* (Thermo Fisher Scientific, Carlsbad, CA, catalog no. C606010). After expression and lysis, protein was purified by Ni^2+^ affinity chromatography and unfolded and refolded to remove residual protein-bound bacterial NA (*26*), followed by cleavage of the 6His tag and purification by affinity and size exclusion chromatography. Protein purity was confirmed by SDS–polyacrylamide gel electrophoresis, and an absorbance ratio at 260 to 280 nm of ≈0.55 was measured, confirming the absence of NA. Before biophysical characterization, the proteins were dialyzed exhaustively in the working buffer [10.1 mM Na_2_PO_4_, 1.8 mM KH_2_PO_4_, 2.7 mM KCl, and 10 mM NaCl (pH 7.40)]. Final protein concentrations were determined by refractometry.

The oligonucleotide T10 (TTTTTTTTTT) was purchased from Integrated DNA Technologies (Skokie, IL), purified by high-performance liquid chromatography (HPLC), and lyophilized. N_LRS_ peptides were purchased from ABI Scientific (Sterling, VA), purified by HPLC, examined by matrix-assisted laser desorption/ionization for purity and identity, and lyophilized.

### Analytical ultracentrifugation

SV experiments were carried out in a ProteomeLab XL-I analytical ultracentrifuge (Beckman Coulter, Indianapolis, IN) as previously described (*65*). Briefly, samples were loaded in cell assemblies comprising charcoal-filled Epon double-sector centerpieces of 3- or 12-mm pathlength and sapphire windows. The cells were temperature equilibrated at 20°C in an AN-60 TI rotor, followed by acceleration to 55,000 rpm. Depending on the solution composition of the samples, data were acquired with Rayleigh interference optics and absorbance optics at 230, 260, and/or 280 nm. Radial profiles were plotted with GUSSI (*66*). Calibration was carried out as described in (*65*). Standard protocols were followed to calculate the sedimentation coefficient distribution *c*(*s*) for each dataset using the software SEDFIT (https://sedfitsedphat.nibib.nih.gov/software) (*67*). Sedimentation coefficients were converted to standard conditions based on compositional partial-specific volumes of 0.732 and 0.717 ml/g for the N_LRS_ peptide and N-FL, respectively. Model-based hydrodynamic predictions were calculated using HYDROPRO (*68*). Sedimentation and diffusion coefficients were combined to calculate molecular weights via the Svedberg equation $M(1-\bar{v}\rho )=(s/D)\mathrm{RT}$ (*65*). For self-association analysis, the integrated weight-average sedimentation coefficients were assembled into isotherms and fitted jointly with CD data by GMMA (*69*). Hydrodynamic nonideality was estimated to be *k*_S_ = 10 ml/g.

### Dynamic light scattering

Autocorrelation data were collected in a NanoStar instrument (Wyatt Technology, Santa Barbara, CA). One hundred–microliter samples were inserted into a 1-μl quartz cuvette (WNQC01-00, Wyatt Instruments), using excess sample to minimize the impact of evaporation in the observation chamber. Laser light scattering was measured at 658 nm at a detection angle of 90°. For the temperature scans, a ramp rate of 1°/min was applied with 5-s data acquisitions and averaging three replicates for each temperature point. Data were collected and processed by using Dynamics 7.4 (Wyatt Technology) and SEDFIT.

### Circular dichroism

CD spectra were acquired in a Chirascan Q100 (Applied Photophysics, UK). N_LRS_ samples were measured in 0.1 or 0.2 mm pathlength cells, dependent on concentration, with 1-nm steps and 1-s integration time, averaging three acquisitions and subtracting buffer backgrounds. For temperature scans, data were acquired in 1-nm intervals with integration times of 0.5 s, without repeats, applying a temperature ramp rate of 1°C/min, followed by analysis using the Global3 software (Applied Photophysics).

The concentration dependence of θ_MRE_(222 nm) was modeled globally with the s_w_ isotherm derived from SV, using the GMMA software SEDPHAT (version 6.1) (*69*). In the GMMA model, molar MRE values for complex species were assumed to be different from that of the monomer. For clarity, best-fit cumulative association equilibrium constants for monomer-*n*-mer equilibria were converted to effective dissociation constants as *K*_D_^*^ = *K*_A_^[1/(*n* − 1)].

The TFE dependence of the MRE at 222 nm was fit with a two-state model. The maximum helicity at 222 nm based on a 37–amino acid peptide was determined as θ_H_ = −35,900 deg cm^2^ dmol^−1^, that of a random coil was taken as θ_RC_ = −1500 deg cm^2^ dmol^−1^, and the number of residues in helical conformation was estimated as 37(θ − θ_RC_)/(θ_H_ − θ_RC_) (*70*, *71*).

### Optical microscopy imaging of in vitro liquid-liquid phase separated condensates

The in vitro phase separation assays were performed as described previously (*21*). Samples of N protein in 10.1 mM Na_2_PO_4_, 1.8 mM KH_2_PO_4_, 2.7 mM KCl, 10 mM NaCl, 10% and polyethylene glycol (pH 7.40) at room temperature were transferred onto a 35-mm glass-bottom dish (catalog no. part no: P35G-1.5-20-C, MatTek). Condensates from LLPS were imaged within 30 to 40 min or a few hours, as indicated. Images were acquired on a Nikon Ti-E microscope equipped with a 100×1.49 numerical aperture oil objective lens (LIDA light engine, Lumencor, Beaverton, OR) and recorded with a Prime 95B camera (Teledyne Photometrics) with a pixel size of 110 nm. Images were background-subtracted and contrast-enhanced using MATLAB (Mathworks, Natick, MA).

### Mutational landscape and sequence alignment

Mutation data were based on consensus sequences of SARS-CoV-2 isolates submitted to the GISAID (*55*) and downloaded on 10 August 2022 as preprocessed metadata file by the Nextstrain team (nextstrain.org) (*72*). As described previously (*21*), only high-quality sequences based on multiple criteria evaluated in Nextstrain analysis were included in a set of 3.83 million genomes, and a threshold of 10 observations of any mutation was used to filter adventitious sequencing errors. The Wuhan-Hu-1 isolate (GenBank QHD43423) was used as the ancestral reference. Alignment of SARS and related betacoronavirus sequences was carried out with COBALT at NLM (*73*), and highlights for similar residues were taken from ESPript (*74*).

### Structure prediction

Structures of the N_LRS_ peptide and FL protein were predicted using ColabFold (*51*), which is an extension of AlphaFold2 and AlphaFold-multimer to predict protein complexes. Graphics were created using ChimeraX (*75*). The peptide’s sequence contains a single stretch of the form *axxacxc* (*c*: charged; *a*: apolar) common to parallel, left-handed coiled-coil heptad repeats. Coiled-coils were identified using Socket2 (*76*).

### MD simulations

Dimers, trimers, and tetramers were considered, each in the predicted coiled-coil arrangement. To avoid conformational bias in the prediction, to better mimic the oligomerization process in solution and to lay the basis for systematic comparative analyses of mutations, we proceeded as follows: monomers (ancestral and mutants) were first simulated at near-experimental conditions; their structures (snapshots after equilibration) were then arranged in the predicted oligomer configurations by aligning (Ca-rmsd minimization) the sequence 6-25. Two models of the N_LRS_ can be created a priori, one extracted from the AlphaFold2 prediction of the full-length protein (fig. S5A) and another one from the ColabFold prediction of the isolated 37-residue sequence (fig. S5B). Both contain a central helix flanked by disordered segments, but the degree of helicity and confidence score differs slightly. The first model was studied previously (*21*), and the second model was simulated here. Over the course of the dynamics, both models converged essentially to the same degrees of helicity and qualitative features. All the simulations were carried out in the isothermal-isobaric ensemble, at 20°C and 1 atm, at [NaCl] ≈150 mM and pH 7. After standard protocols of heating and equilibration, a productive phase of 30 ns was conducted, and analysis was performed over the last 20 ns. Soft definitions of H-bond/salt-bridge and hydrophobic/nonpolar interactions are based on a distance (δ) criterion between donor and acceptor atoms (δ_AD_ < 4 Å) and side chain carbon atoms (δ_CC_ < 4.8 Å), respectively. CD spectra were calculated using the software DichroCalc (https://comp.chem.nottingham.ac.uk/dichrocalc/). For additional details on the simulation setup, see (*21*).

### Statistical analysis

Biophysical data analyses were carried out on the basis of least-squares fits and Fisher *F*-statistics. To avoid noise amplification in sedimentation analysis, maximum entropy regularization was applied.

## Notes

View/request a protocol for this paper from *Bio-protocol*.
