# Supplementary material for: A conserved oligomerization domain in the disordered linker of coronavirus nucleocapsid proteins
Source: Sci Adv. 2023 Apr 5;9(14):eadg6473. doi: 10.1126/sciadv.adg6473 (PMC10075959; doi:10.1126/sciadv.adg6473)
Supplement: Supplementary file 1 — Figs. S1 to S17 Table S1 [file sciadv.adg6473_sm.pdf]

Science Advances
AAAS

Supplementary Materials for

A conserved oligomerization domain in the disordered linker of coronavirus nucleocapsid proteins

Huaying Zhao *et al.*

Corresponding author: Peter Schuck, schuckp@mail.nih.gov

Sci. Adv. **9**, eadg6473 (2023)
DOI: 10.1126/sciadv.adg6473

This PDF file includes:

Figs. S1 to S17
Table S1

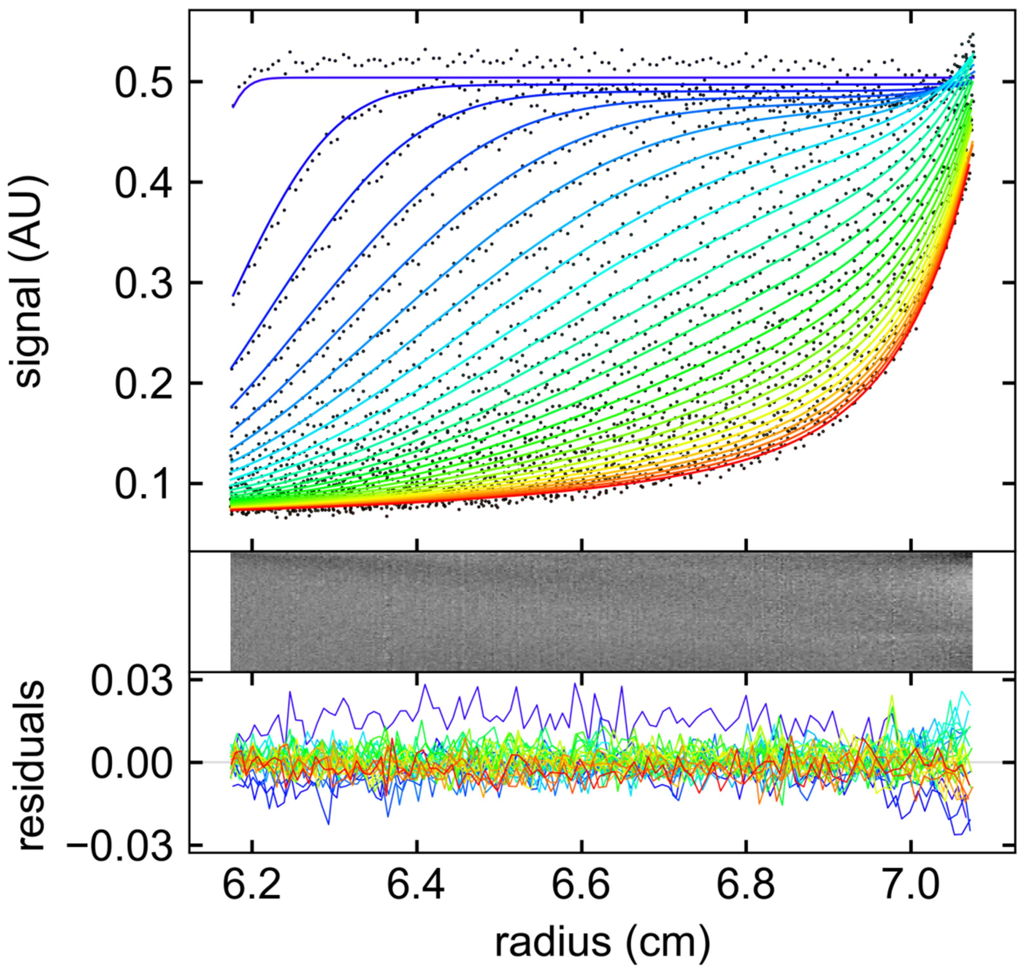

Supporting figure S1

Typical sedimentation boundaries of N_{LRS}. Top: Shown are absorbance profiles at 230 nm acquired at a rotor speed of 55,000 rpm, plotted in time-intervals of 54 min. Every third data point is shown (dots) along with the radial profile from the best-fit *c*(*s*) model (lines) with time indicated by color temperature. The derived sedimentation coefficient distribution *c*(*s*) is shown in Fig. 2. Middle and Bottom: Residuals bitmap and residuals overlay plot of the fit. The figure was created with the software GUSSI (kindly provided by Dr. Chad Brautigam).

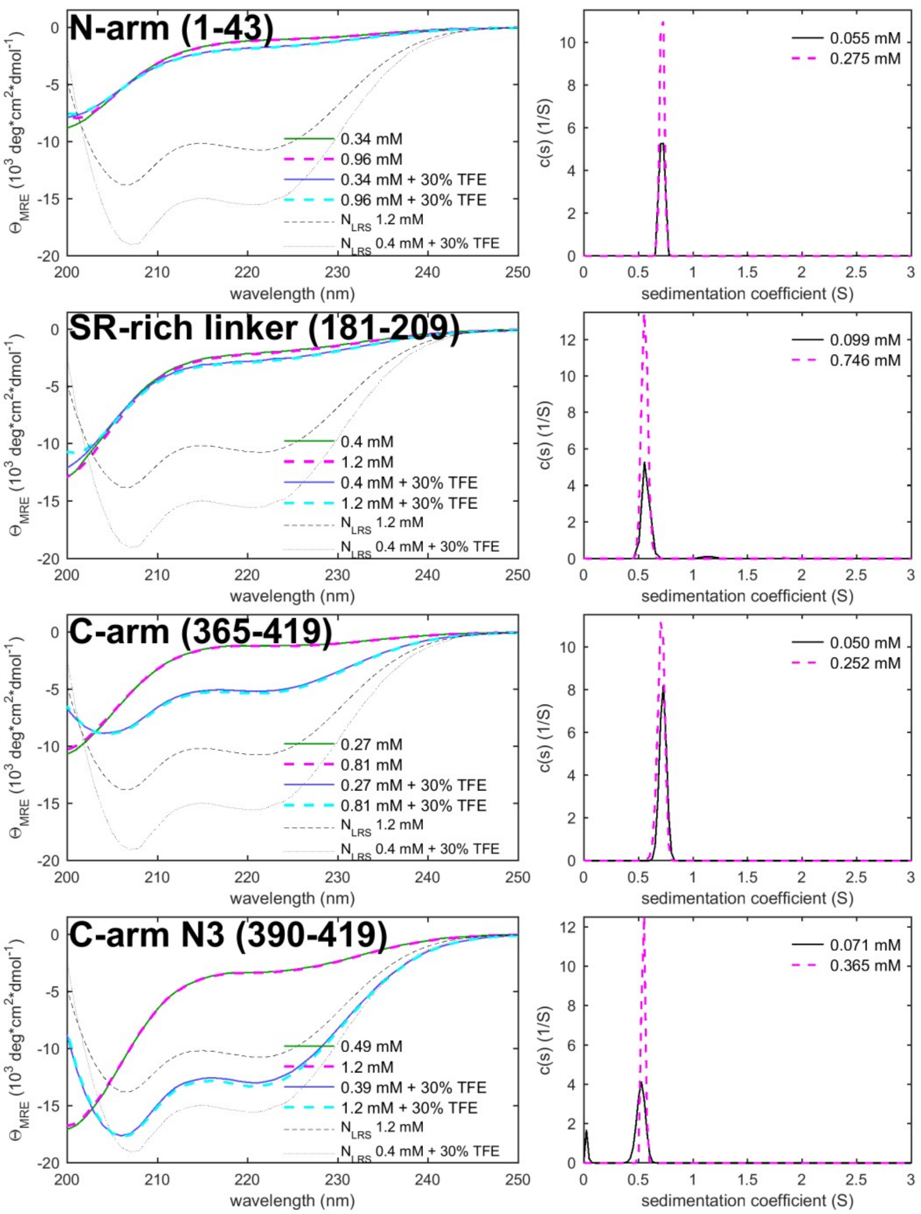

Supporting figure S2

Absence of self-association and helical structure in other IDRs of N-protein.
Left Column: For the different IDRs, the left panels show the concentration dependence of peptide CD spectra in the absence (green solid and magenta dashed lines, virtually overlapping), and the same concentrations in the presence of 30% TFE (blue and cyan dashed lines, virtually overlapping). Data are plotted in mean residue ellipticity units, which makes the data from different sized peptides directly comparable, and comparable with the N_{LRS} spectra at high concentrations (black dashed lines) and medium concentration in 30% TFE (black dotted lines).
Right Column: For the same peptides the sedimentation coefficient distributions *c*(*s*) obtained in SV experiments at low and high concentrations. The peak heights and areas signify different sample concentrations, whereas different *s*-values for different peptides are a result of their different molecular weight. All *s*-values are consistent with the monomeric state of the respective peptides.

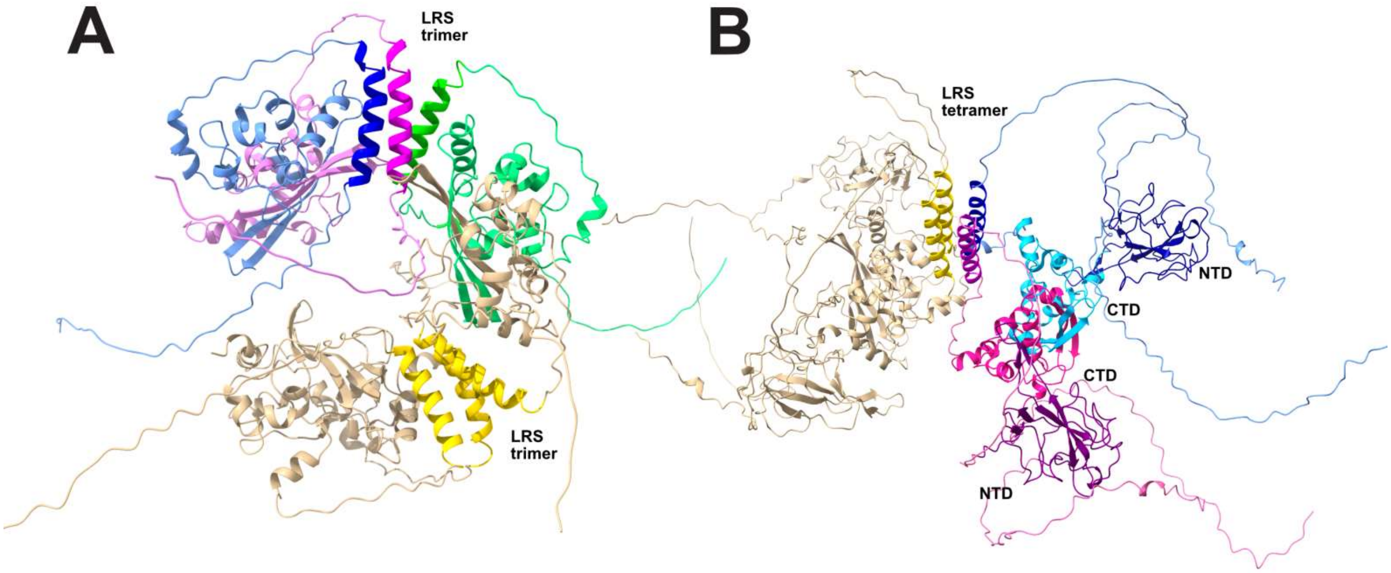

Supporting figure S3

Oligomeric configurations of FL-N-protein predicted by ColabFold.
(A) Hexamer of six linker-CTD constructs (N:181-364) with three CTD dimer contacts and two LRS trimers. Only three chains are highlighted in blue, red, and green tones for clarity. Truncation was required due to limitations in ColabFold GPU memory and computation time.
(B) Tetramer of full-length N-proteins with pairwise CTD dimer contacts and LRS tetramer contacts. Two chains in the symmetric tetramer are colored in blue and red tones, respectively. While the CTD of each chain is engaged in dimeric contacts, the LRS regions form a parallel coiled-coil. The NTD of each chain is linked by disordered regions and in a random position not in contact with any other domain.

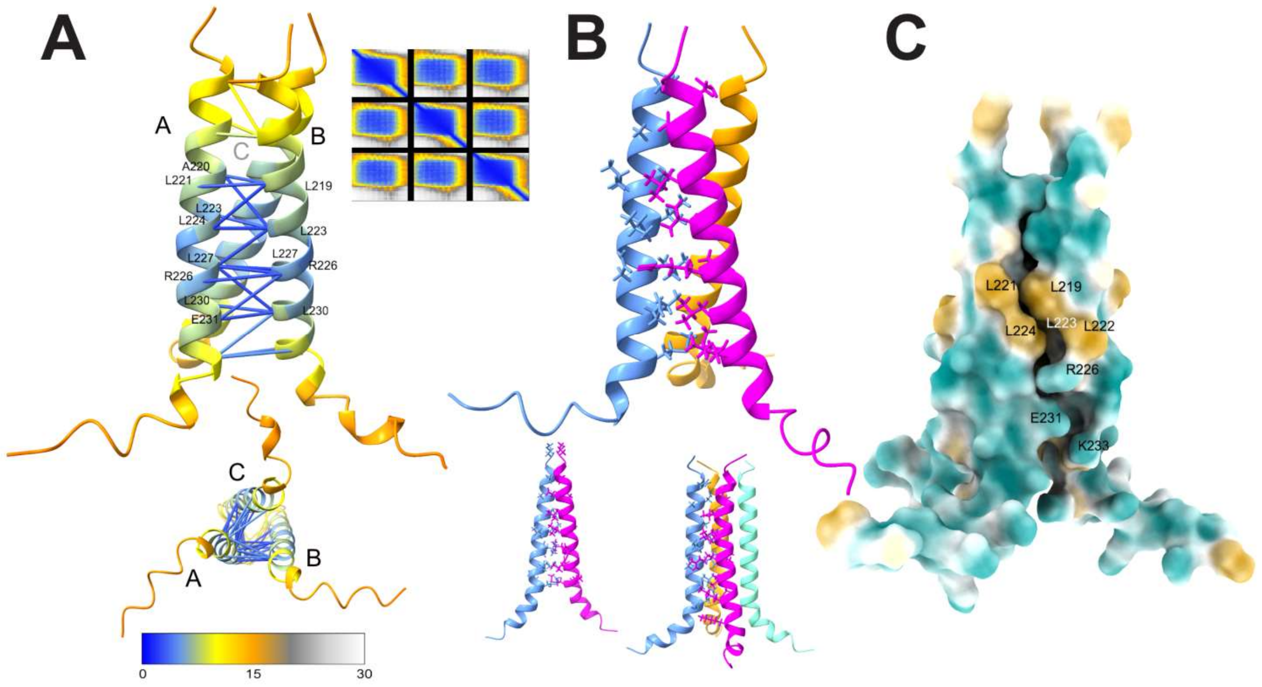

Supporting figure S4

Oligomeric structures of the LRS peptide predicted by ColabFold. Good prediction confidence was achieved, highest for the trimer with maximum pLDDT score of 86.8, closely followed by a dimer (84.9) and the tetramer (76.7).

(A) Predicted alignment error (PAE) of the trimer N_{LRS} peptide, depicting high confidence predictions in blue. The PAE matrix shows high-confidence intermolecular contacts in the off-diagonal. Highlighted in the structure as pseudo-bonds are contacts (residues within 3.5 Å) of helix A and B (side view) and A and B/C (top view). The residues in contacts with highest confidence are labeled.

(B) Predicted structure of the trimer, dimer, and tetramer with highlighted side-chains of residues of the blue and magenta helix that are within 3.5 Å. The oligomeric structures are close to symmetric with regard to their conformation and intermolecular contacts.

(C) The trimer surface with color rendered by hydrophobicity shows shape complementary of chains. Residues highly protected from mutations are labeled.

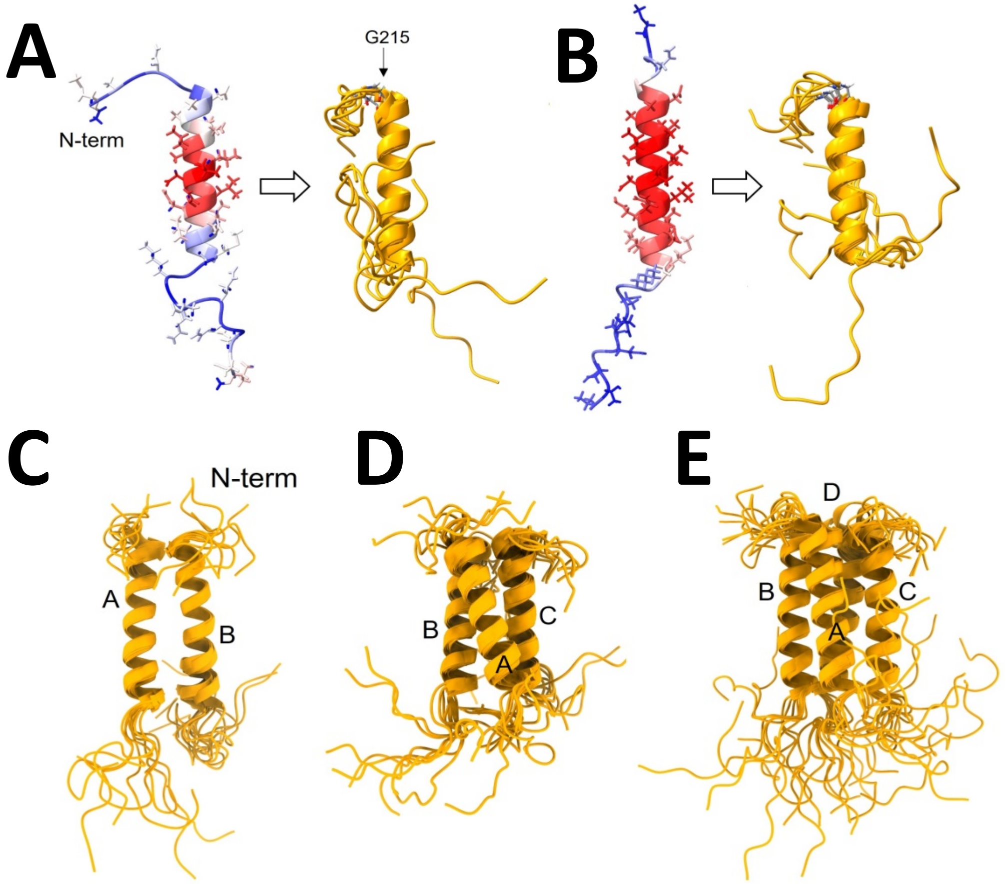

Supporting figure S5

Snaphots along the MD simulations for N_{LRS} monomer and oligomers.

(A,B) On the left are shown N_{LRS} monomer extracted from the AlphaFold2 model of the full-length N-protein (A) or the ColabFold model of the isolated 37-residue sequence (B), respectively. On the right are snapshots along the 30-ns MD simulations in solution.

(C-E) Snapshots along the 30-ns dynamics simulations of the dimer (C), trimer (D), and tetramer (E).

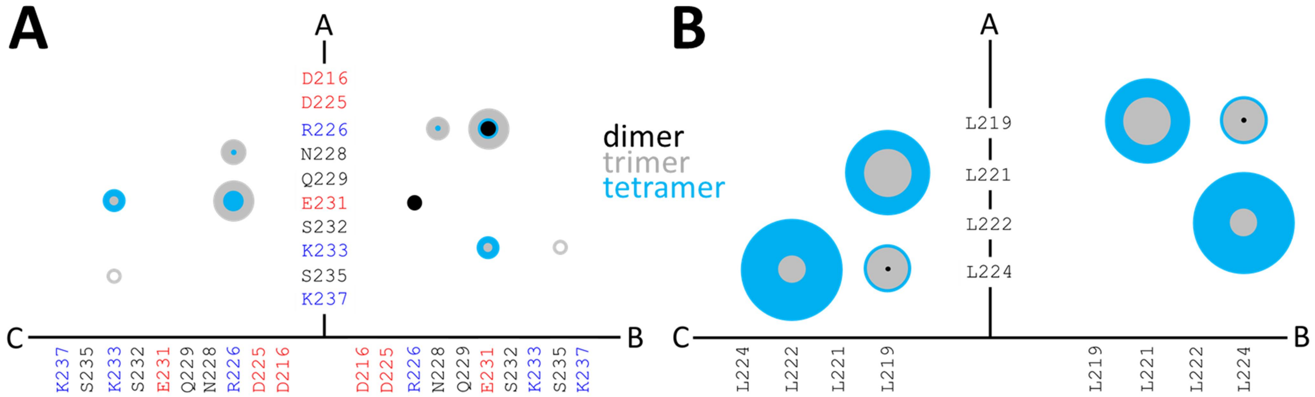

Supporting figure S6

Statistical analysis of MD simulations comparing interactions in different N_{LRS} oligomers.
Helix labels A, B, and C are as in fig. S5. For the different interactions, the size of each solid circle is proportional to the frequency of interaction throughout the oligomers' dynamics and can be considered a proxy for its strength. Open circles are transient interactions (size not proportional to the frequency) that may become relevant upon suitable mutations.
(A) Statistics of electrostatics/H-bond interactions.
(B) Hydrophobic interactions of the residues on the stabilizing surface patch. This patch is not a major stabilizing force for dimers, but it is significant in higher-order oligomers.

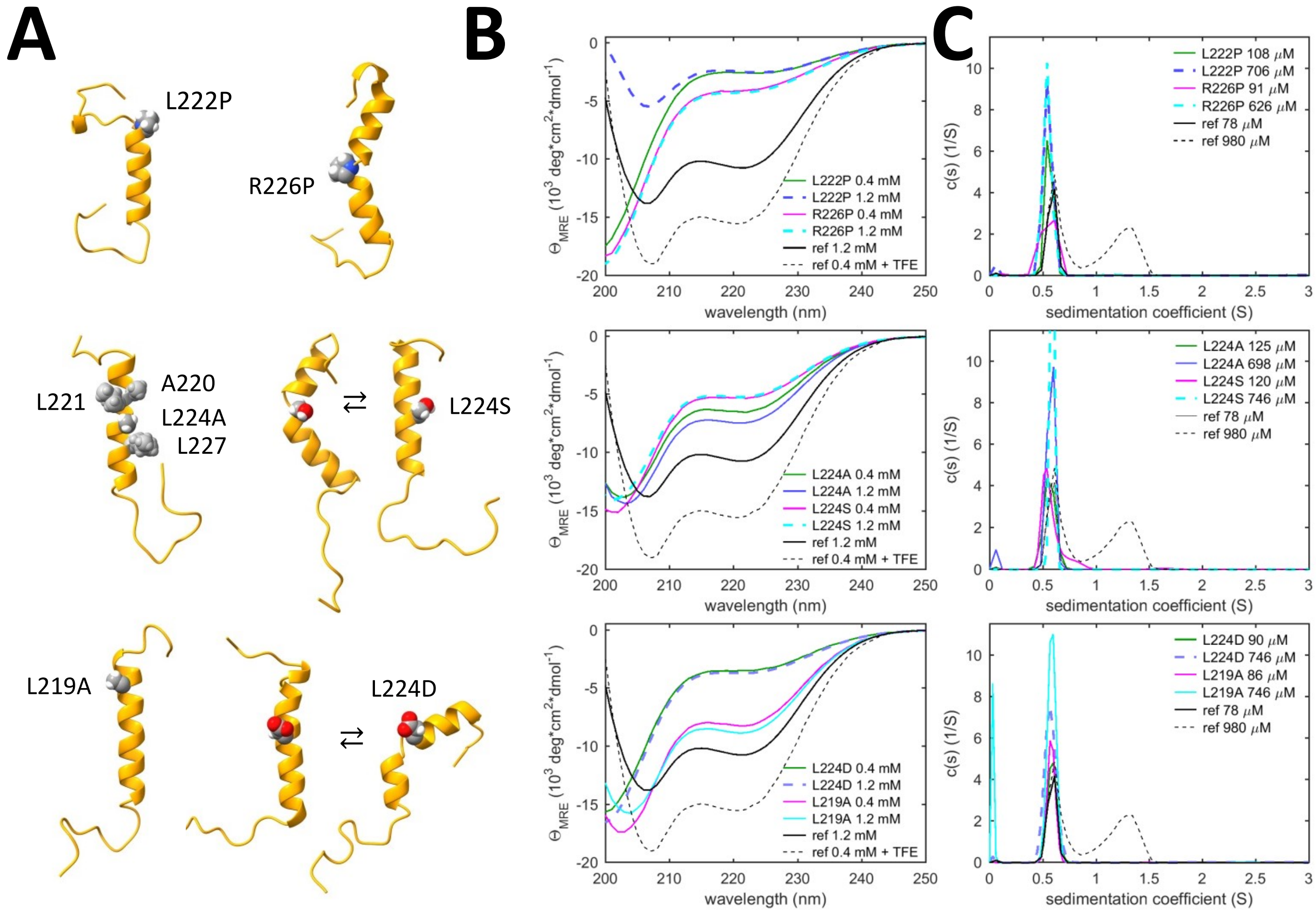

Supporting figure S7

N_{LRS} mutants abrogating helix formation and self-association.

(A) Snaphots of MD simulations. Top row: Peptides N_{LRS}:L222P and N_{LRS}:R226P were introduced to disrupt the helix. The simulations show that P222 disrupts the cohesiveness of the surface hydrophobic patch in all the oligomers, which is effectively kept in place in the reference N_{LRS} peptide by leucine. The hinge, clearly observed in the monomer, repositions the upper portion of the helices so that A217 and A218 in the oligomers take the role of L222, partially compensating for the loss of the hydrophobic interaction with L222. Less acute is the hinge introduced by P226, as the interactions between surrounding residues and their sizes prevent complete helix bending. However, the loss of the salt bridge with E231 in the oligomers is not compensated, which likely affects complex stability, particularly in the trimer, where it is stronger. Middle row: Three mutations were introduced at position L224 to probe the role of this critical leucine on the surface hydrophobic patch: in N_{LRS}:L224A, the smaller size of alanine weakens the hydrophobic forces that keep adjacent helices in place. Polar or charged residues at position 224 also destabilize the monomer due to the weakened hydrophobic forces mediated by the bulky/branched leucine in N_{LRS}. In the oligomers, N_{LRS}:L224S disrupts the hydrophobic interactions without any compensating force and destabilizes the helix creating transient kinks; distortions of a-helixes are common even in the absence of prolines. Bottom row: N_{LRS}:L224D behaves similar to N_{LRS}:L224S, although it also interacts electrostatically with R226. Mutant N_{LRS}:L219A shows little difference in the monomer's behavior, but alanine has a deleterious effect on the stability of the hydrophobic cluster in the oligomers, similar to that in N_{LRS}:L219A. In particular,

the smaller alanine disengages L221, leading to a measurable opening of the upper portion of the oligomers and residues D216 more exposed to the solvent.

(B) CD spectra of the same mutants at different concentrations. As a reference, spectra for N_{LRS} without mutations are shown at 1.2 mM without TFE, and at 0.4 mM with 30% TFE (solid and dashed black lines). Most mutants show little or no concentration-dependence, with exception of N_{LRS}:L222P.

(C) Concentration dependence of sedimentation coefficient distributions for the same mutants. For reference, the distribution of N_{LRS} without mutations at 78 µM and 980 µM are shown as solid and dashed black lines. None of the mutants display self-association.

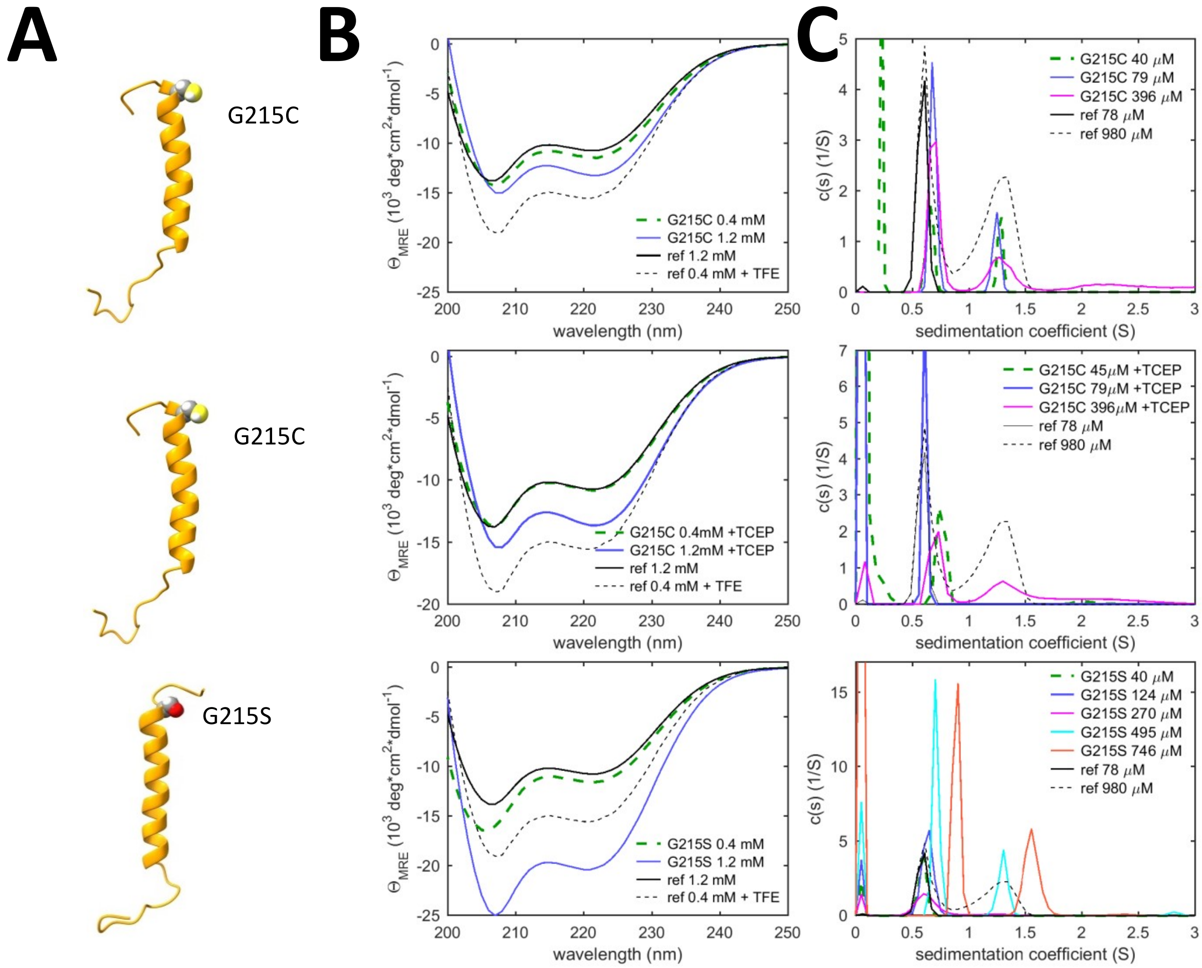

Supporting figure S8

N_{LRS} mutants enhancing helix formation and self-association.

(A) Snaphots of MD simulations. Top and Middle row: In the peptide N_{LRS}:G215C the C215 side chain redirects the tethered portion of the linker upward. Bottom row: The peptide N_{LRS}:G215S displays no structural or dynamic differences to N_{LRS}:G215C, neither in the monomer nor in the oligomers.

(B) CD spectra of the mutants at different concentrations. The middle row displays experimental results in the presence of 1 mM TCEP reducing disulfide bonds partially crosslinking N_{LRS}:G215C peptides. As a reference, spectra for N_{LRS} without mutations are shown at 1.2 mM without and at 0.4 mM with 30% TFE (solid and dashed black lines).

(C) Concentration dependence of sedimentation coefficient distributions for the same mutant. At similar concentrations, all of the mutants exhibit stronger self-association than the reference peptide N_{LRS} lacking any mutations. Supplementing the working buffer with 1 mM TCEP results in fewer oligomers for N_{LRS}:G215C, consistent with the formation of disulfide bonds in addition to reversible self-association in the absence of reducing agent, as described previously (20). For reference, the distribution of N_{LRS} without mutations at 78 μM and 980 μM are shown as solid and dashed black lines. Assuming the mechanism of self-association and linked folding to be identical, from the similarity of CD spectra and SV profiles of 0.4 mM N_{LRS}:G215C and 1.2 mM N_{LRS} it is possible to estimate the free energy enhancement to be -1.3 kcal/mol.

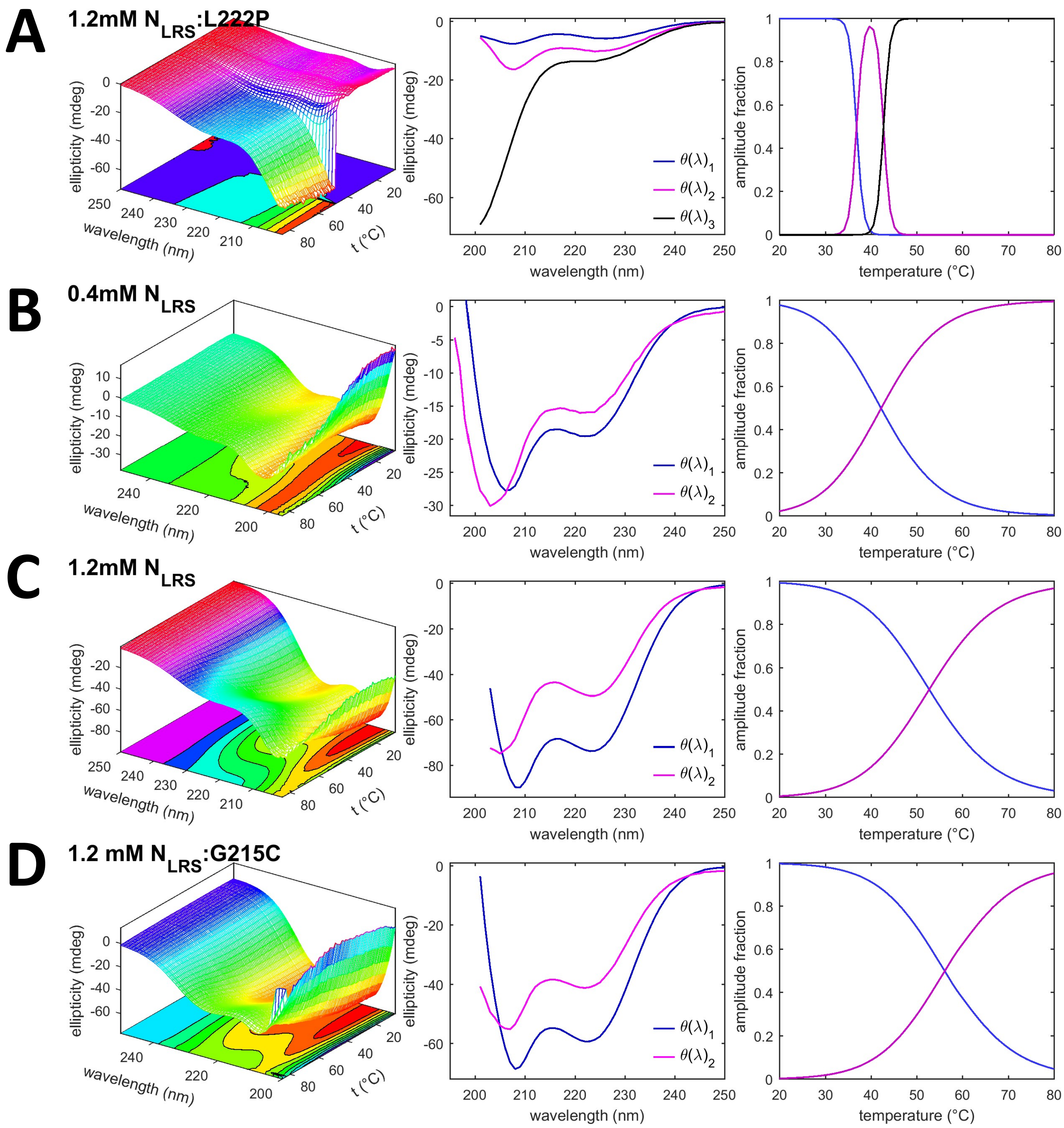

Supporting figure S9

Temperature-dependent folding of N_{LRS} and enhancing and abrogating mutants. Raw CD data as a function of wavelength and temperature (Left Column) are decomposed into spectral components (Middle Column) and their corresponding temperature-dependent amplitudes (Right Column). All measurements are carried out with identical optical pathlengths. However, normalization to mean-

residue ellipticity is not possible due to partial sedimentation of particles formed above the transition temperature leading to reduced concentrations in the light path.

(A) The mutant N_{LRS}:L222P was shown to abrogate helix formation and self-association. In the temperature scan it shows little helicity below a transition at 36.5 °C to a helical state, which unfolds into a largely disordered state at 42.5 °C.

(B,C) The reference peptide N_{LRS} exhibits concentration-dependent helicity. From the temperature scan a transition can be discerned at 42 °C (0.4 mM) and 52.5 °C (1.2 mM) to a state with greater magnitude of the ratio of signals at 208 nm and 222 nm, and slight shift of the first minimum to lower wavelength (at 1.2 mM, minimum is at 208 nm at low *versus* 202 nm at high temperature). This is indicative of a state at higher temperature that is still largely helical but with higher disordered fraction.

(D) The mutant N_{LRS}:G215C has been shown to enhance helicity and self-association. The temperature-dependent CD spectra show a similar transition as the reference N_{LRS} peptide, but at a higher temperature of 55.8 °C. From the slightly higher ratio of $\theta(208)/\theta(222)=1.34$ compared to the reference peptide ($\theta(208)/\theta(222)=1.17$ for N_{LRS} at 1.2 mM), as well as the smaller shift in the first minimum (206 nm at high temperature), the high-temperature state appears to maintain a greater degree of folding than N_{LRS}.

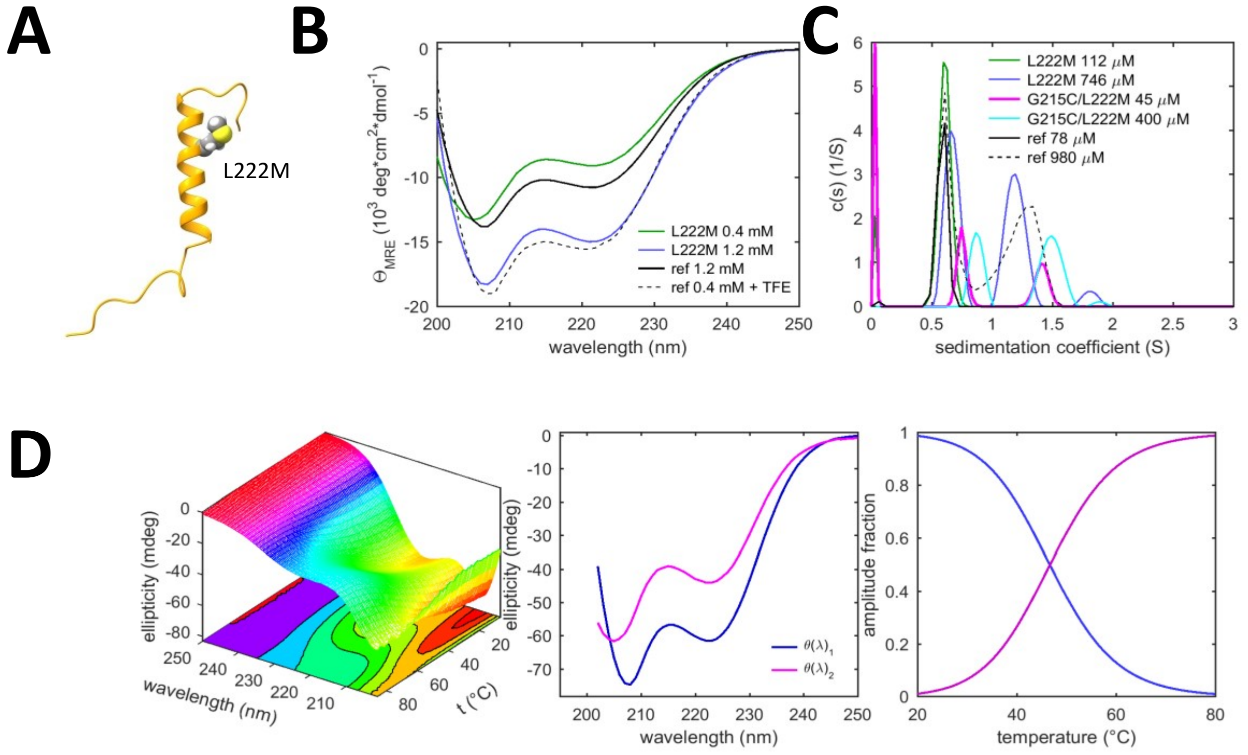

Supporting figure S10

Properties of the naturally occurring mutant L222M in N_{LRS} .

(A) Snapshot of the structure of N_{LRS}:L222M in MD simulations.

(B) CD spectra of N_{LRS}:L222M at different concentrations. As a reference, spectra for N_{LRS} without mutations are shown at 1.2 mM without TFE and at 0.4 mM with 30% TFE (solid and dashed black lines).

(C) Concentration dependence of sedimentation coefficient distributions for N_{LRS}:L222M and the double mutant N_{LRS}:G215C/L222M. For reference, the distribution of N_{LRS} without mutations at 78 μM and 980 μM (solid and dashed black lines).

(D) Raw CD data of 1.2 mM N_{LRS}:L222M as a function of wavelength and temperature (left) are decomposed into spectral components (middle) and their corresponding temperature-dependent amplitudes (right). From the temperature scan a transition can be discerned at 46.4 °C.

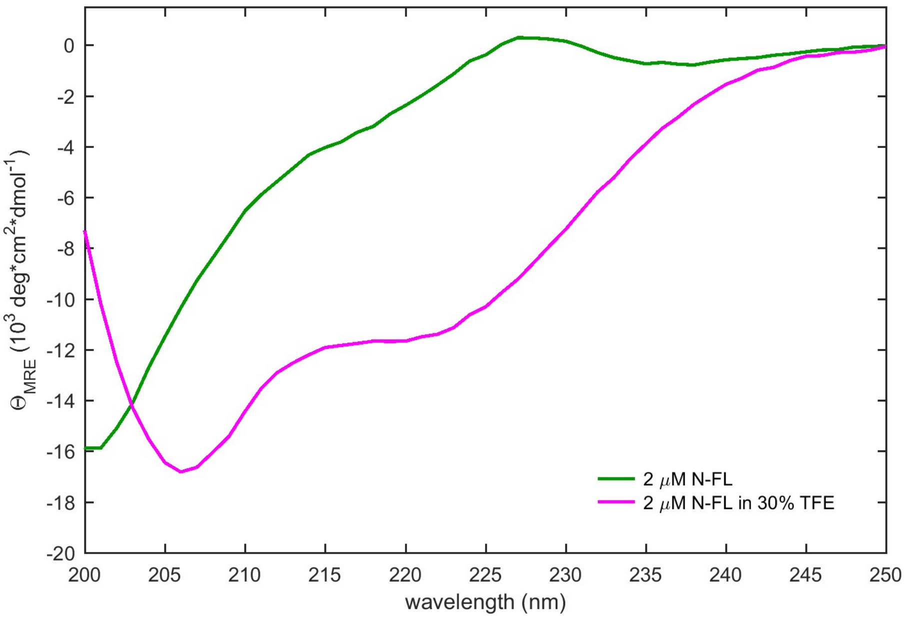

Supporting figure S11

The effect of TFE on FL-N. CD spectra of FL-N in working buffer (green) and supplemented with 30% TFE (magenta).

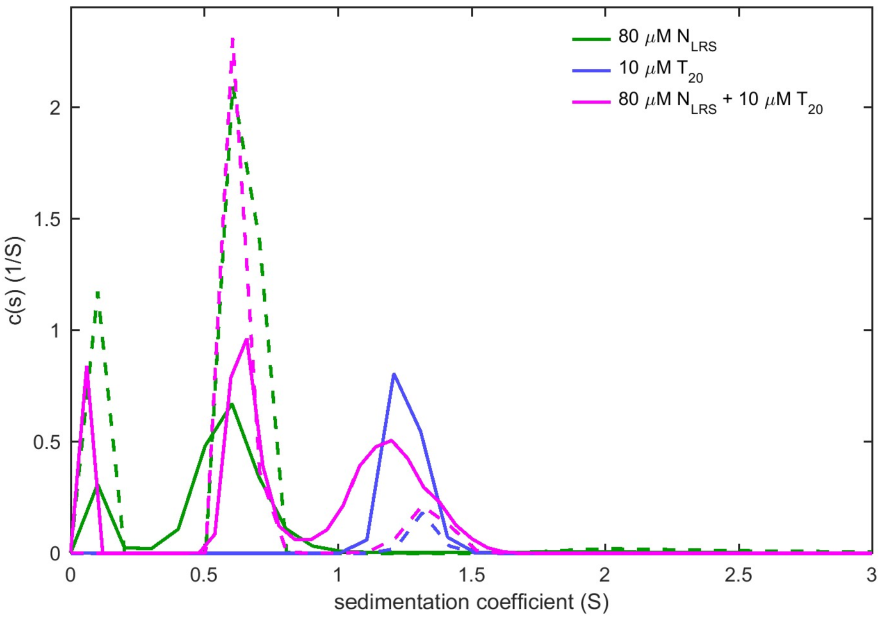

Supporting figure S12

Probing NA binding of $\mathbf{N_{LRS}}$. SV experiment of N_{LRS} (green), T_{20} (blue), and a mixture (magenta) under conditions where strong binding of NA to FL-N is observed, and with the large molar excess of N_{LRS} . Shown are sedimentation coefficient distributions $c(s)$ for data acquired at 230 nm (solid lines) and with the interference optical detection system (dashed lines). The absorbance signal is dominated by T_{20}, whereas the interference signal is dominated by the peptide. 1:1 complexes would be expected to sediment between 1.5 and 2.5 S. The absence of absorbance $c(s)$ signal in this range places a lower limit for K_D of a potential N_{LRS}-T_{20} interaction in the mM range.

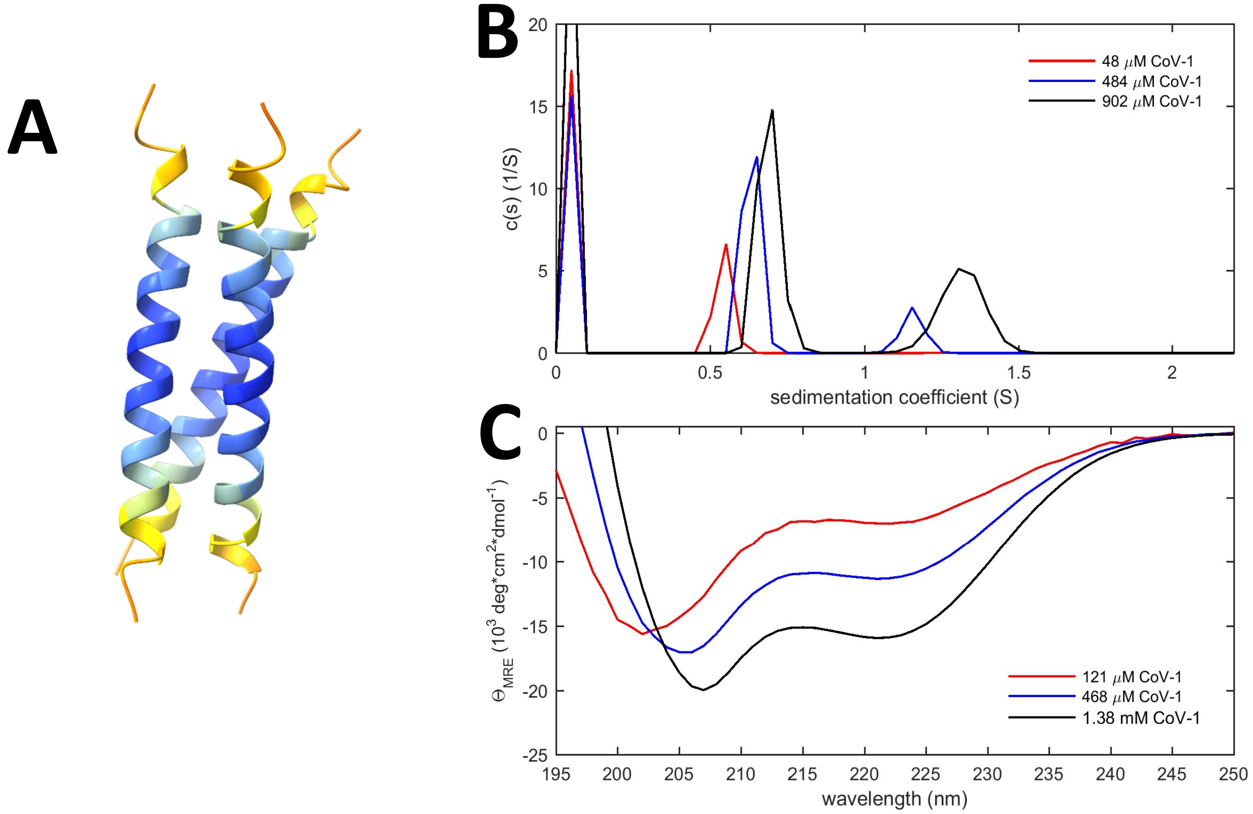

Supporting figure S13

Predicted trimeric structure and concentration-dependent linked self-association/folding of SARS-CoV-1 nucleocapsid LRS peptide.

(A) Predicted structure of trimeric SARS-CoV-1 N-peptide 211-242 from ColabFold.

(B) Experimental sedimentation coefficient distributions of the peptide measured at different concentrations shows oligomerization at higher concentrations.

(C) Mean residue ellipticity spectra at a range of concentrations show assembly-linked helical folding.

\

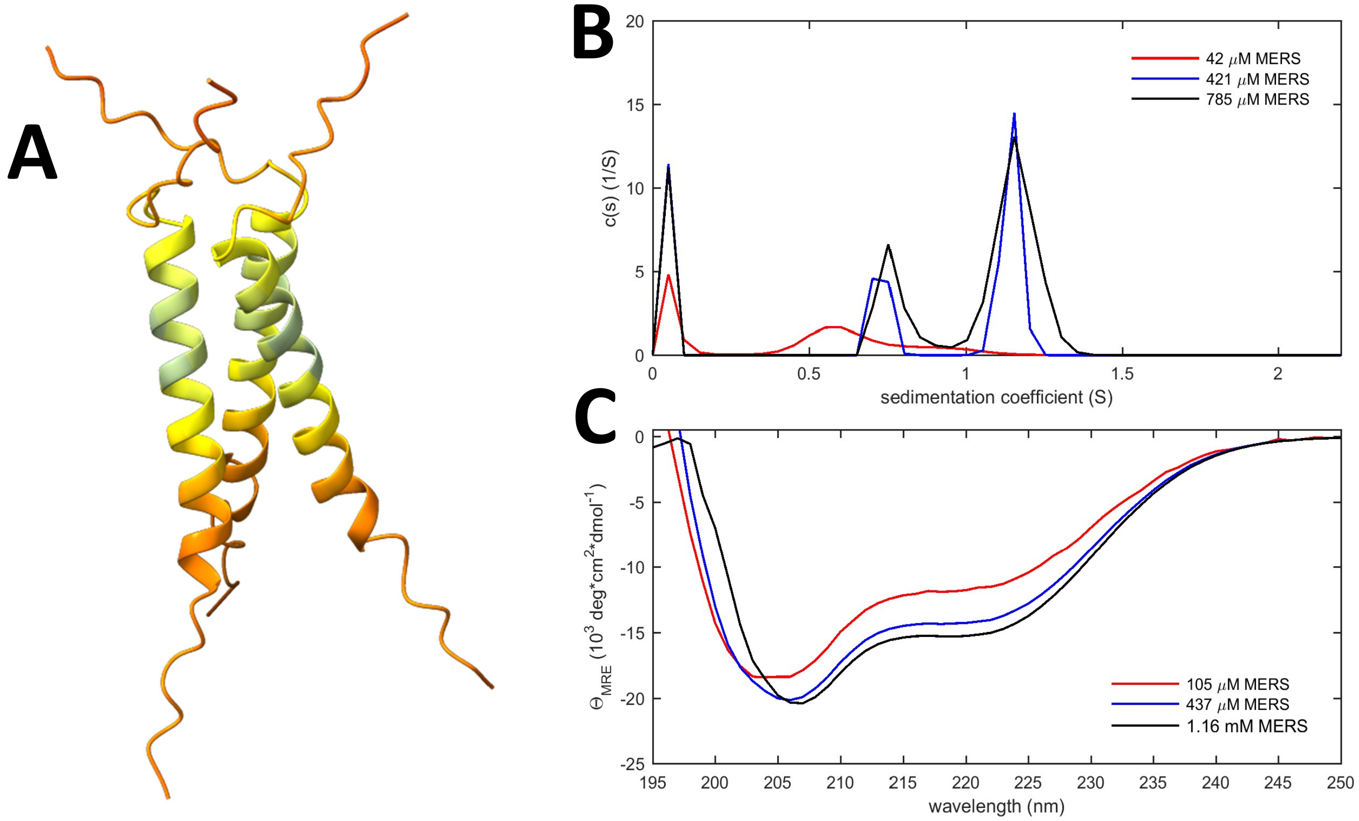

Supporting figure S14

Predicted trimeric structure and concentration-dependent linked self-association/folding of MERS nucleocapsid LRS peptide.

(A) Predicted structure of trimeric MERS N-peptide 197-238 from ColabFold.

(B) Experimental sedimentation coefficient distributions of the peptide measured at different concentrations shows oligomerization at higher concentrations.

(C) Mean residue ellipticity spectra at a range of concentrations show assembly-linked helical folding.

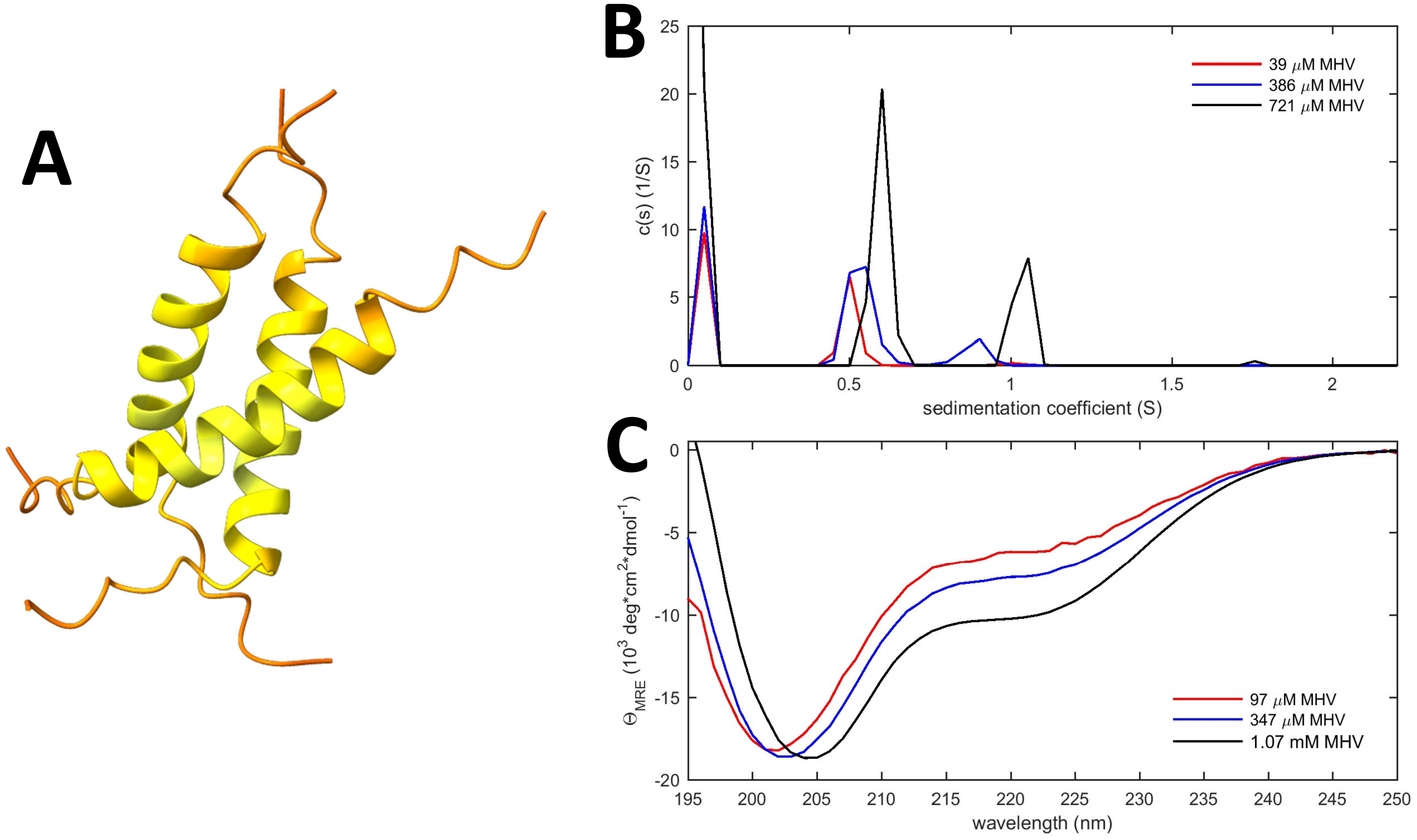

Supporting figure S15

Predicted trimeric structure and concentration-dependent linked self-association/folding of MHV nucleocapsid LRS peptide.

(A) Predicted structure of trimeric MHV N-peptide 223-255 from ColabFold.

(B) Experimental sedimentation coefficient distributions of the peptide measured at different concentrations shows oligomerization at higher concentrations.

(C) Mean residue ellipticity spectra at a range of concentrations show assembly-linked helical folding.

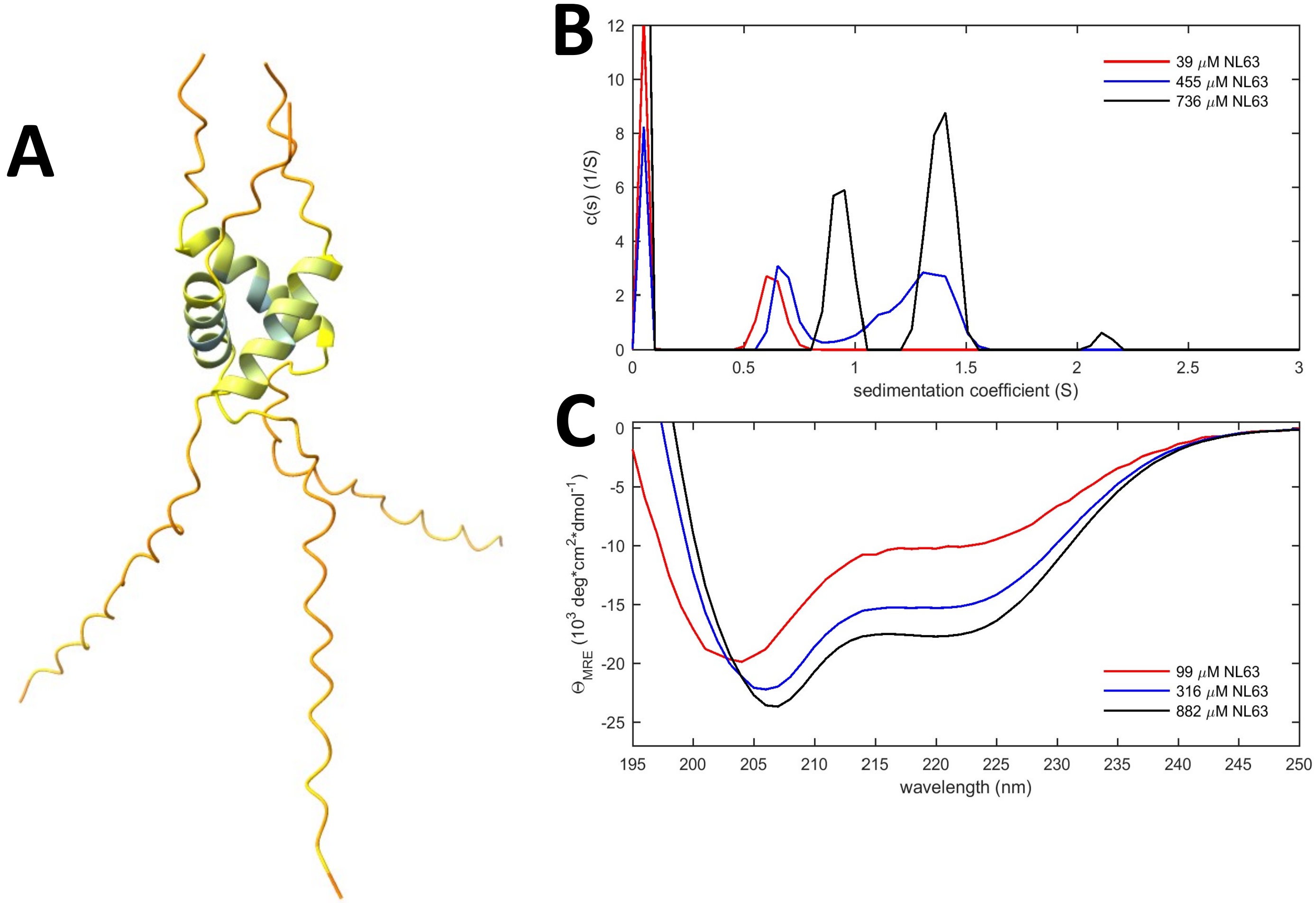

Supporting figure S16

Predicted trimeric structure and concentration-dependent linked self-association/folding of NL63 nucleocapsid LRS peptide.

(A) Predicted structure of trimeric NL63 N-peptide 174-210 from ColabFold.

(B) Experimental sedimentation coefficient distributions of the peptide measured at different concentrations shows oligomerization at higher concentrations.

(C) Mean residue ellipticity spectra at a range of concentrations show assembly-linked helical folding.

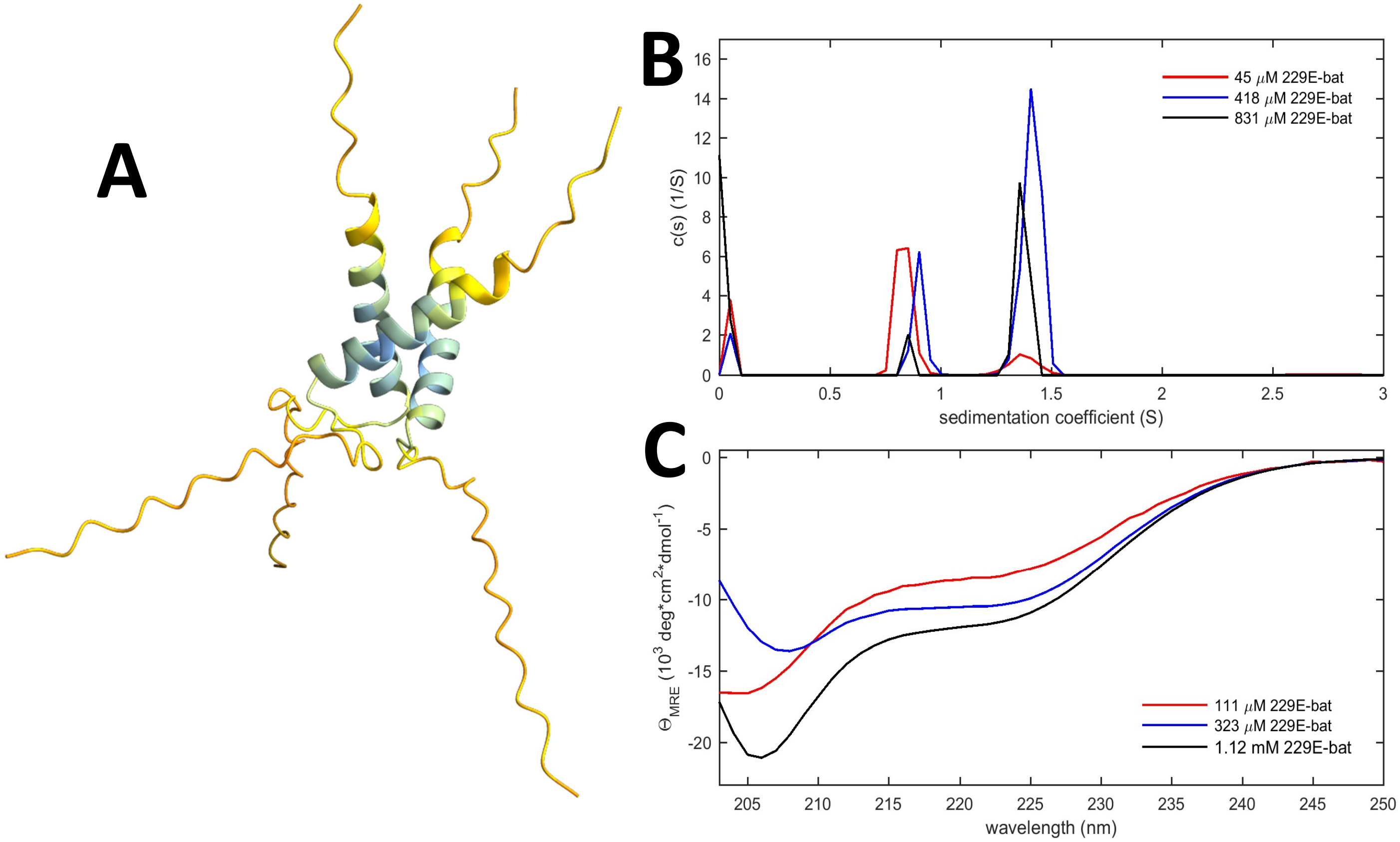

Supporting figure S17

Predicted trimeric structure and concentration-dependent linked self-association/folding of 229E-related bat coronavirus nucleocapsid LRS peptide.

(A) Predicted structure of trimeric N-peptide 170-221 from ColabFold.

(B) Experimental sedimentation coefficient distributions of the peptide measured at different concentrations shows oligomerization at higher concentrations.

(C) Mean residue ellipticity spectra at a range of concentrations show assembly-linked helical folding.

Table S1. Coronavirus LRS peptide properties

virus	nucleocapsid accession #	mass (kDa)	K^*_D (mM)	ΔGtrimer (kcal/mol)	range	sequence
SARS-CoV-2	YP_009724397	3.8	0.81	-8.4	210-246	MAGNGGDAALALLLLDRLNQLESKMSGKGQQQQGQTV
SARS-CoV-1	P59595.1	3.3	0.50	-8.863	211-242	MASGGGETALALLLLDRLNQLESKVSGKGQQQ
MERS	YP_009047211.1	4.3	0.11	-10.8	197-238	RGTSPGPSGIGAVGGDLLYLDLLNRLQALESGKVKQSQPKVI
MHV	NP_045302.1	3.5	0.80	-8.4	223-255	RQPASTVKPDMAEEIAALVLAKLGKDAGQPKQV
NL63	Q6Q1R8.1	3.8	0.14	-10.5	174-210	TRSDSNQSSSDLVAAVTLALKNLGFDNQSKSPSSSGT
229E-related bat	APD51511.1	5.7	0.29	-9.7	170-221	NERLPKSNSKQSNQDDIMAAVAAALEKLGFERPNDASQPQKKQNKGTPKPSR
